# The Andean-Amazonian and Mesoamerican Bioeconomy: A new paradigm for productivity and well-being

**DOI:** 10.1371/journal.pone.0345710

**Published:** 2026-06-23

**Authors:** Jorge Leon Quiroga-Canaviri, Carlos Alberto Zuniga-González, Carlos Ricardo Menéndez Gámiz, Adelfa Patricia Colón-García, Ruth Maria Cruz Puerto

**Affiliations:** 1 Posgrados en Agronomía (UPA) y de Ciencias del Desarrollo (CIDES) de la Universidad Mayor de San Andrés (UMSA), Universidad Tecnológica de Centro América (UNITEC) en Honduras, La Paz, Bolivia; 2 National Autonomous University of Nicaragua, Leon, Center for Research on Bioeconomy and Climate Change, Area of Knowledge in Agricultural and Veterinary Sciences, Specific Directorate of Agroecology and Agribusiness, León, Nicaragua; 3 Universidad Autónoma Chapingo – CIESTAAM, Estado de México, Chapingo, Texcoco, México; 4 Universidad Nacional Autónoma de Honduras, Carrera de Economía Agrícola UNAH-Atlántida, La Ceiba, Hondura; Veracruzana University: Universidad Veracruzana, MEXICO

## Abstract

Traditional Total Factor Productivity (TFP) metrics often overlook biophysical limits and the depletion of natural capital. This study proposes an alternative paradigm: the Andean-Amazonian Bioeconomy (AAB), which integrates Georgescu-Roegen’s Law of Entropy and ancestral knowledge into an expanded production function. By applying the Malmquist Productivity Index and a Fixed Effects (FE) panel data model, we analyzed a dataset (1995–2024) from six Latin American countries. This approach utilizes the Human Development Index (HDI) as the primary proxy for social welfare. The methodology addresses country heterogeneity and multicollinearity, significantly enhancing the model’s explanatory power to an Adjusted R^2^ of 0.695. Our findings reveal a ‘Biocultural Paradox’: where conservation, often viewed as a cost by traditional income-based metric, emerges as vital investment. Our model demonstrate that the preservation of the biocultural fund- represented here as Biocultural Savings [S]- is a positive and highly significant predictor of social well-being (*β* = 0.685, *p* < 0.01), alongside a positive impact from the indigenous population index (*β* = 0.015, *p* < 0.1), suggesting that cultural identity plays a foundational role in regional resilience. We conclude that achieving ‘Vivir Bien’ (Living Well) requires a shift toward indicators that prioritize the biocultural fund -the stock of ancestral knowledge and biodiversity- over mere resource flow. This study provides a scientifically rigorous framework for sustainability policies across the Global South.

## 1. Introduction

The prevailing economic approach has proven insufficient to address systemic instabilities, specifically climate change, biodiversity loss, and deepening wealth inequality [1,2]. This gap is reflected in conventional performance metrics like Total Factor Productivity (TFP), which fail to account for the entropic degradation of natural resources or social equity [3]. The core critique targets the treatment of the economy as an isolated system, a premise demystified by Georgescu-Roegen [4] via the Law of Entropy. This law posits that the economic process is an irreversible entropic flow, transforming low-entropy resources into high-entropy waste [5]. The Andean-Amazonian Bioeconomy (AAB) mitigates this degradation by prioritizing ‘fund’ maintenance—such as biodiversity and ancestral knowledge—over rapid material throughput, thereby slowing the rate of entropic increase and decoupling social well-being from exhaustive resource depletion.

The AAB emerges as a theoretical and practical response to this void, adapting traditional structures to principles such as cosmic–territorial balance (the equilibrium between human production and ecosystem regeneration), community reciprocity (non-market resource exchange), and Buen Vivir (a multidimensional well-being index) [6,7]. As argued by Menéndez Gámiz et al. [8], this paradigm recognizes ancestral knowledge as a fundamental element of well-being [9]. The scientific contribution of this paper is twofold: it establishes a theoretical framework for reinterpreting productivity beyond the limitations of TFP, and it provides empirical evidence of how biocultural assets drive regional development.

To bridge these ancestral concepts with measurable outcomes, we adopt Amartya Sen’s capability approach [10,11] as the appropriate lens to define well-being not as income, but as the expansion of human freedoms and territorial resilience. To empirically validate these theoretical propositions, we utilize the Human Development Index (HDI) within a Fixed Effects (FE) model, ensuring methodological rigor by addressing country-specific heterogeneity and potential multicollinearity in the Andean-Amazonian context.

## 2. Literature review: The Andean–Amazonian Bioeconomy paradigm

The literature reveals a growing but fragmented field of bioeconomy studies—split into bio-resource, bio-technological, and bio-ecological perspectives—that has yet to fully integrate ecological and social imperatives [12,13]. This fragmentation often stems from the tension between “Green Growth” (which seeks to decouple GDP from environmental impar through technology) and “Degrowth”(which argues for a structural reduction of throughput) narratives. Within this context, the Andean–Amazonian Bioeconomy (AAB) emerges as a distinct paradigm that redefines traditional economic frameworks by integrating ancestral knowledge with modern biophysical science-specifically thermodynamics and landscape ecology—to address the metabolic requirements of regional development [12].

While neoclassical attempts to internalize environmental costs, such as the Dasgupta Review’s approach to “Natural Capital,” have made progress, they often remain tied to a weak sustainability focus where natural assets are potentially substitutable. In contrast, the AAB is rooted in Georgescu-Roegen’s [4] entropy-based critique, moving beyond the mechanical assumptions of the Cobb–Douglas function. As noted by Quiroga-Canaviri [14], this requires a transition toward a “flow-fund” model that accounts for the irreversible degradation of energy and matter. To operationalize this transition in the Andean-Amazonian context, the Jach’a Qh’anax model (Aymara for “Great Light of Knowledge”) provides a bridge, translating ancestral biocultural funds into measurable indicators of social and ecological resilience [11,15].

The AAB framework operationalizes these principles through three core pillars: (i) Bioculture, which treats ancestral knowledge as a non-depletable human capital fund; (ii) Bio-territoriality, which views biodiversity as a dynamic asset rather than an inert factor; and (iii) Bio-governance, which establishes the institutional framework for community-led resource management [16,17]. By redefining these inputs, the AAB provides the empirical structure necessary to transition from theoretical entropy to the productivity metrics explored in this study.

The study encompasses a diverse and representative sample of Latin American economies, including Bolivia, Ecuador, Guatemala, Honduras, Mexico, and Nicaragua. This selection allows for a robust comparative analysis between the Andean-Amazonian core and the Mesoamerican corridor. These regions were integrated into a unified analytical framework due to their shared reliance on biocultural capital and their common challenges regarding climate change and sustainable productivity.

## 3. Materials and methods

### 3.1. Conceptual definitions

To assist the reader in navigating the empirical framework of this study, this section defines the highly technical econometric and bioeconomic terms utilized in our analysis:

Exogenous: Refers to factors, variables, or shocks that originate outside the immediate economic or biological model. These variables influence the internal dynamics of the system (such as well-being or productivity) but are not influenced by the system itself.

Malmquist Productivity Index: A non-parametric method used to measure changes in Total Factor Productivity (TFP) over time. It allows for the decomposition of productivity growth into two distinct components: efficiency change (catching up to the frontier) and technological change (shifts in the frontier itself).

Production Frontier: The geometric or mathematical boundary representing the maximum possible output or well-being reachable by an economy or territory given a specific set of inputs (such as biocultural capital) and the current state of technology.

Technological Change: Improvements in processes, innovations, or the integration of ancestral knowledge that shifts the production frontier upward, allowing for higher levels of output or welfare using the same or fewer resources.

### 3.2. Construction of composite indicators and reproducibility

The empirical analysis is based on a balanced panel data set covering six Latin American countries (Bolivia, Ecuador, Guatemala, Honduras, Mexico, and Nicaragua) for the period 1995–2024 [18]. While the model is rooted in the Andean-Amazonian paradigm, these countries were selected to test the scalability of the AAB framework across diverse Latin American socio-ecological contexts, from equatorial rainforests to Mesoamerican dry forests.

Data were harmonized from specific databases, including the World Bank’s World Development indicators, (Agricultural production and land use data), and the RICYT (Science and technology indicators). To ensure comparability, all monetary variables were adjusted to constant 2015 US dollars. Missing values, which accounted for less than 5% of the observations, were treated using linear interpolation. This method was selected because, given the low percentage of missingness, it preserves long-term trend without introducing the artificial volatility that more complex imputation might generate in a 30-year span.

To ensure reproducibility and operationalize the Andean-Amazonian Bioeconomy framework, composite indicators were constructed using raw data from the sources detailed in Table 1, the complete data matrix used for the studied countries is available in (S2 File). To ensure numerical robustness and avoid the “zero-value” problem in Data Envelopment Analysis (DEA), all variables were normalized using Min-Max method (Eq 1) and subsequently scaled to a range of [0.001, 1]. The use of 0.001 as a floor is a standard *epsilon* in efficiency models to prevent division-by zero error without significantly altering the sensitivity of the efficiency scores.

**Table 1:** Operationalization of variables, definitions, and data sources for the Andean-Amazonian Bioeconomy model (1995–2024).

Variable	Definition and Unit of Measurement	Relation to Bioeconomy Pillar	Source
Household Consumption	Total market value of all goods and services, including durable products, purchased by households. (Constant LCU).	Community Consumption (C): Measures resilience and human development capacity.	World Bank / Central Banks
Biocultural Savings	Economic proxy for collective investment in natural assets and non-monetary community resource management.	Biocultural Savings (S): Represents the preservation of natural/cultural capital.	Authors’ calculation based on World Bank data [19,20]
Indigenous Population	Percentage of the total population identifying as indigenous or belonging to an ethnic minority.	Symbolic Capital (K): Proxy for the density of ancestral knowledge and cultural identity.	Nacional Censuses / World Bank
Forest Cover	Percentage of land area covered by forests and strategic ecosystems for biodiversity.	Ecological Balance (E): Indicator of ecosystem health and regenerative capacity.	Global Forest Watch / Terra-i
Political and Social Participation	Index of civil society engagement and collective social action intensity.	Reciprocity (A): Measures non-monetary mutual support and social fabric.	V-Dem Institute / World Bank
TFP_AAB_	Total Factor Productivity of the Andean-Amazonian Bioeconomy (Malmquist Index).	Outcome Variable: Measures the efficiency of the regenerative model.	Authors’ estimation [11,21,19]


$$\begin{array}{c} X_{norm}=\text{0}.\text{0}\text{0}\text{0}\text{1}\;+\frac{X-X_{min}}{X_{max}-X_{min}}x\;(\text{1}-\text{0}.\text{0}\text{0}\text{1}) \end{array}$$ (1)

### 3.3. Analytical framework: From traditional TFP to bioeconomic productivity (TFP_AAB_)

We propose an expanded production function that integrates nature and culture as endogenous structural components [14]. Unlike the traditional Cobb-Douglas model, the Bioeconomic Productivity TFP_AAB_, is defined as (Eq. 2):


$$\begin{array}{c} \;{TFP}_{AAB}\;=f\;(N,B,T,I) \end{array}$$ (2)

Where N is Natural Capital, B is Bioculture, T is Bioterritoriality, and I is Bioinformation [11]. Within this framework, we explicitly operationalize the flow of entropy: CO_2_ emissions are treated as ‘High-Entropy Undesirable Outputs,’ while **Biocultural Savings (S)** are categorized as ‘Low-Entropy Inputs.’

The Biocultural Savings (S) represent the net national income adjusted by socio-environmental factors according to the following adjustment (Equation 3):


$$\begin{array}{c} S=\;NNI-(CC+DN+DC) \end{array}$$ (3)

(Where NNI is Net National Income, CC is Carbon Costs, DN is Depletion of Natural funds, and DC is Degradation of Cultural capital). This metric accounts for the preservation of biocultural funds, where the conservation of indigenous territories acts as a non-monetary “saving” (BK) that prevents future restoration costs [14], the mathematical boundaries are described in (S1 File).

### 3.4. Empirical estimation: The Malmquist Productivity Index

To evaluate efficiency changes, the Malmquist Productivity Index between periods t and t + 1 is employed [11,21]. The index is decomposed into Technical Efficiency Change (EC), representing improvements in resource use, and Technological Change (TC), representing shifts in the production frontier due to biocultural innovation (Eq. 4):


$$\begin{array}{c} {\overset{t,t+\text{1}}{\underset{\text{0}}{M}}(x^{t},y^{t},x^{t+\text{1}},y^{t+\text{1}})=[\frac{\overset{t}{\underset{\text{0}}{D}}(x^{t+\text{1}},y^{t+\text{1}})}{\overset{t}{\underset{\text{0}}{D}}(x^{t},y^{t})}\cdot \frac{\overset{t+\text{1}}{\underset{\text{0}}{D}}(x^{t+\text{1}},y^{t+\text{1}})}{\overset{t+\text{1}}{\underset{\text{0}}{D}}(x^{t},y^{t})}]}^{\text{1}/\text{2}} \end{array}$$ (4)

where:

$x^{t}\;y\;x^{t+\text{1}}$ are the input vectors in periods t and t + 1,

$y^{t}\;y\;y^{t+\text{1}}$ are the output vectors in periods t and t + 1,

$\overset{t}{\underset{\text{0}}{D}}$ (x, y) is the output-oriented distance function in period t.

Note on Interpretation: In this study, TC is specifically interpreted as biocultural innovation. Unlike conventional models where TC reflects exogenous industrial progress, here it represents the shift in the production frontier driven by the integration of ancestral management and regenerative practices that expand biophysical limits. The analysis was conducted using **R software (v. 4.3.1)**, utilizing the *nonparaeff* and *plm* packages for efficiency and panel estimation, respectively, the econometric routine was executed using R Studio syntax (S3 File).

### 3.5. Econometric specification: Fixed effects (FE) model

To bridge these theoretical propositions with measurable outcomes, we implement a **Fixed Effects (FE) Panel Data Model** [22]. To validate the impact of bioeconomic productivity on welfare, we adopt **Amartya Sen’s Social Welfare Function (SWF)**, defined as, where is mean income (Real GDP per capita) and is the **Gini Complement** or “Equity Index” [23,24]. This specification was selected over Random Effects following a Hausman-White Robust Standard Errors (Equation 5):


$$\begin{array}{c} {Ln\;W}_{it}=\alpha_{i}+\beta_{\text{1}}ln({TFP}_{BAA,\;it}){+\beta }_{\text{2}}{ln\;(S}_{it})+\beta_{\text{3}}{\;ln\;(K}_{it})+\in_{it} \end{array}$$ (5)

Where $W_{it}$ is the social welfare index: ${TFP}_{BAA,\;it}$ is the bioeconomic productivity; S_it_ represents the Biocultural Savings; K_it_ is the Indigenous Identity index; $\alpha_{i}$ represents the country-specific fixed effects; and $\in_{it}$ is the error term [11]. The use of a log-log specification allows the coefficients to be interpreted as elasticities while stabilizing functional variance*.* Subsequently GDP and the Gini coefficient into logarithmic and complementary forms allows (1 – Gini) allow the model to capture constant elasticities and distributive equity. As detailed in Table 2, this alignment is essential for the stability of the long-term equilibrium and the ‘Vivir Bien’ principle. This structural alignment is essential for the stability of long-term subsequent cointegration analysis and the stability of the long-term structural breaks [19,20]. The operationalization of these specific proxies for the Social Welfare Function is summarized in Table 2. By redefining the TFP_AAB_ as a function of entropic efficiency, this framework allows for a quantitative transition from traditional growth models to a bio-centric productivity measure consistent with ‘Vivir Bien’ [23–25].

**Table 2:** Operationalization of the social welfare function: Indicators and statistical treatment.

Variable	Proxy Usage	Source	Statistical Note
Welfare (W)	HDI (Human Development Index)	UNDP / World Bank	Used to estimate β and α elasticities.
Income (y)	Real GDP per capita	World Bank	Log-transformed for elasticity estimation.
Equity (1-G) TFP_AAB_	Gini Complement Bioeconomic Productivity	World Bank Authors’ Estimation	Measures the efficiency of distribution. Independent variable linking bioeconomy to welfare.

This approach controls time-invariant characteristics, such as geographic scale and institutional baselines, significantly reducing omitted variable bias.

### 3.6. Model robustness and diagnostic test

The selection of the FE ‘Within’ model was scientifically justified by the Hausman specification (*χ2* = 34.52, *p <* 0.01), which rejected the null hypothesis of no correlation between the individual effects and the regressors. To ensure reliability or the results. We performed the **Breusch-Pagan** and **Wooldridge** tests. To address these issues, we utilized **Huber-White robust standard errors**, ensuring a consistent covariance matrix despite country-level heterogeneity [26–28]. The stationarity of the logged series was verified through the Levin-Lin-Chu (LLC) and Im-Pesaran-Shin (IPS) unit root tests, confirming that the variables are integrated of order zero (I (0).

## 4 Results

### 4.1. Analysis of bioeconomic productivity (TFP_AAB_)

The results of the Malmquist Index, adjusted for the Andean-Amazonian Bioeconomy (TFP_AAB_), reveal a generally upward trend in resource efficiency across the region, with five out of six countries exceeding the unity threshold (Table 3). The region is characterized by a “Biocultural Paradox,” where productivity growth depends on balancing technical advancement with the preservation of ancestral funds [25,29].

**Table 3:** Summary of bioeconomic productivity performance (Mean 1995–2024).

Country	Mean (TFP-AAB)	Technical Change (tc)	Pure Efficiency (pech)	Scale Efficiency (sech)	Entropic Interpretation
Bolivia	1.042	1.057	1.050	0.938	Improved productivity via technical advancement despite scale inefficiencies.
Ecuador	0.997	1.044	1.000	0.954	Stable productivity; technical progress offset by scale inefficiencies.
Guatemala	1.088	1.088	1.000	1.000	Productivity growth driven entirely by technical progress at optimal scale.
Honduras	1.030	1.010	1.019	0.999	Balanced growth via internal management improvements and slight technical gain.
México	1.117	1.117	1.000	1.000	Strong productivity growth driven by technical advancement at optimal scale.
Nicaragua	1.221	1.144	1.067	1.000	Highest productivity growth combining technical progress and improved management.

Note: Values > 1 indicate productivity growth; values < 1 indicate decline.

Nicaragua (1.221) and México (1.117) exhibit the highest bioeconomic productivity. For Nicaragua, this growth is a balanced result of high Technological Change (1.144) and improved management efficiency (1.067). In Mexico, the growth is driven entirely by Technical Change (1.117) at an optimal scale. These results suggest successful management of “Biocultural Funds,” where innovation effectively mitigates the biophysical costs of production.

In contrast, Guatemala (1.008) and Bolivia (1.042) exhibit a profile of active transition. In Bolivia, growth is primarily driven by Technological Change (1.057), which compensates for scale inefficiencies (0.938). For Honduras (1.030), the data confirms a resilient fabric where growth is sustained by internal management improvements (1.019) combined with steady technological integration (1.010). Conversely, Ecuador shows average TFP_AAB_ values below unity (0.997) is the only country with a value below unity, reflecting significant entropic pressure where technical progress (1.044) is neutralized by scale inefficiencies (0.954).

This result suggests that the increase in biophysical costs (captured in the undesirable outputs of the model) outweighs the gains in adjusted national income, resulting in a marginal net loss of bioeconomic efficiency over the 30-year period.

**Fig 1:**
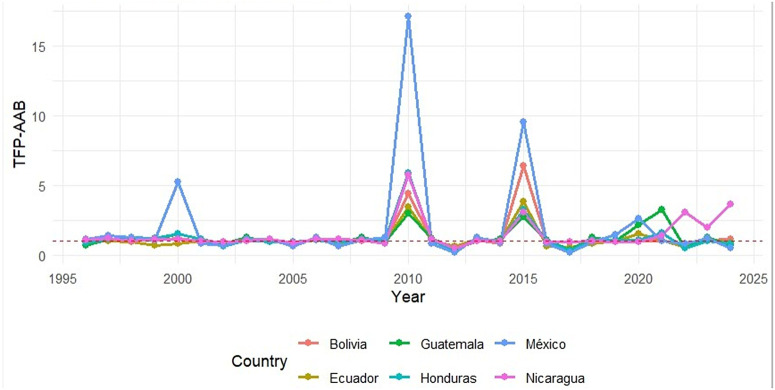
Trends in Andean-Amazonian and Mesoamerican Bioeconomy total factor productivity (TFP-AAB) by country (1996–2024). (The red dashed line represents the equilibrium threshold of productivity where TFP-AAB = 1). The vertical axis represents the annual growth rate; values above the zero-baseline confirm productivity gains, while peaks signify rapid technological integration.

Fig 1 illustrates the annual trajectory of the TFP-AAB for the six countries analyzed over the 1996–2024 period. Visualization reveals significant volatility across all nations, particularly during the early 2000s and around 2010.

Nicaragua demonstrates a resilient upward trajectory, consistently remaining above the equilibrium threshold in the last decade, highlighting a successful integration of biocultural funds. Conversely, Mexico exhibits extreme volatility; while it shows high productivity spikes (e.g., around 2010), these are followed by sharp declines, suggesting a heavy reliance on high-entropy industrial flows that are difficult to sustain within biophysical limits. Ecuador displays a trend of “active stability,” fluctuating narrowly around the unity line, indicating that technical progress is consistently neutralized by inefficiencies in scale or management. Overall, the trends confirm that productivity in the region is characterized by a “Biocultural Paradox,” where sustainable growth depends less on the speed of industrial flow and more on the regenerative capacity of natural and ancestral funds.

### 4.2. Heat map analysis: The entropic flow

**Fig 2:**
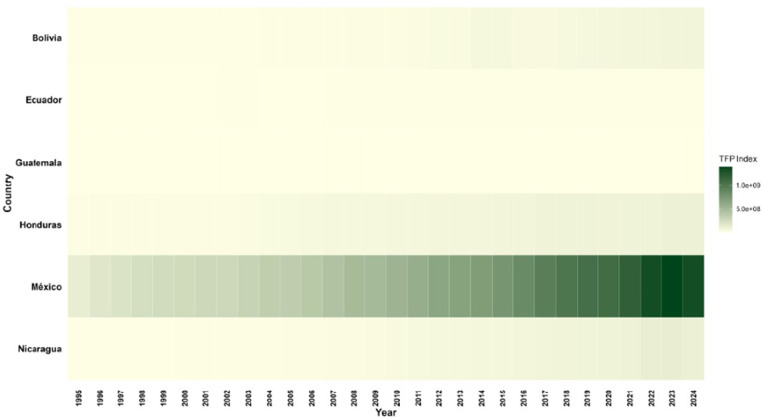
Malmquist Index heat map by country and year. The gradient scale represents the Biocultural Productivity Index (TFP). Dark green cells identify high-efficiency periods (Technological Change > 1), while lighter areas indicate periods of high entropic pressure where efficiency remains stagnant.

Fig 2 provides a visual perspective of the bioeconomic process through the lens of Georgescu-Roegen’s Entropy Law [4]. In this context, a TFP_AAB_ value > 1 (dark green cells) serves as a mathematical proxy for thermodynamic efficiency, representing periods where the system minimizes entropic waste relative to biocultural output.

The intense green peaks represent periods where Technological Change (TC) > 1, interpreted here as biocultural innovation—the internal application of regenerative ancestral knowledge that shifts the production frontier. Conversely, the red cells reveal declines linked to specific biophysical pressures, such as the severe droughts and energy price volatility observed in the region around 2008–2009, which forced systems to increase energy throughput to maintain output. A critical observation is that Efficiency Change (EC) remains near 1.0, suggesting a stable management baseline. From an entropic standpoint, this highlights that the region’s strength lies in continuous biocultural innovation which acts as a regenerative buffer against environmental degradation. [4].

### 4.3. Econometric analysis: From traditional welfare to the AAB paradigm

To validate the impact of bioeconomic productivity, we conducted a regression analysis in two steps. First, a baseline OLS model was used to test the relationship with a general welfare proxy (Table 4, n = 174), where observations with missing HDI components were excluded. For the second stage (n = 180), we utilized a balanced panel through the Fixed-Effects (FE) estimator. The increase in the number of observations was made possible by treating minor missing values in the time series via linear interpolation, allowing the FE model to account for the full longitudinal structure of the six countries over 30 years.

**Table 4:** Preliminary regression: TFP-AAB and proxy Sen’s welfare function.

Variable	Coefficient (β)	Std. Error
TFP-AAB	37.4852	56.5614
Biocultural Savings	0.512***	0.082
Life Expectancy	174.1197***	25.6603
Observations	174 ------------------------------	
R^2^	0.6380	
Adjusted R^2^	0.6280	
F Statistic	99.8899*** (df = 3;170)	

Note: *p < 0.05; **p < 0.01; ***p < 0.001

#### 4.3.1. Statistical validation via fixed effects (FE) model.

The results of the Fixed Effects (FE) model (Table 5) indicate a robust relationship between bioeconomic factors and social well-being (proxied by Life Expectancy). The model explains 71% of the variance (R^2^ = 0.710).

**Table 5:** Fixed effects regression results (Proxy: Life expectancy / well-being).

Variable	Coefficient (β)	Std. Error
Log Biocultural Savings (S)	0.685***	0.224
Biocultural Identity (Index K)	0.015*	0.008
Log Real per capita GDP (y)	3.487***	1.075
Log Ecological Footprint (CO_2_)	−31.3090***	5.174
-----------------------------------------		
Observations	180	
R^2^	0.710	
Adjusted R^2^	0.695	
F statistic	104.068***(df = 4:170)	

Note: *p < 0.1; ** *p* < 0.05; ***p < 0.01.

As shown in Table 5, the Log of Biocultural Savings (S) is positively associated with longevity (*β* = 0.685, *p* < 0.01). Consistent with the log-log specification of the fixed-effects model, this coefficient represents elasticity: a 1% increase in biocultural savings translates into a 0.685% improvement in the welfare proxy (HDI). This finding is central to our argument, as it quantifies the efficiency with which ancestral knowledge and biological conservation are converted into huma development. The Biocultural Identity Index (K) also shows a positive, though marginally significant, impact (*β* = 0.015, *p* < 0.1), suggesting that cultural density supports health outcomes. Conversely, the Log Ecological Footprint (CO_2_) exhibits a substantial negative association (*β*=−31.309, *p* < 0.01). In a log-log framework, such a high magnitude highlights that well-being in these territories is hyper-sensitive to environmental degradation. This relationship acts as a systemic ‘brake’ on development, where entropic pressure and the carbon footprint severely offset the potential gains from traditional industrial throughput, reinforcing the need for the bioeconomic transition proposed in this study.

## 5. Discussion

The results of the Fixed Effects (FE) model (Table 5) provide a robust validation of our core hypotheses, confirming that biocultural variables are structural drivers of long-term development in the region [30]. By controlling for time-invariant country-specific heterogeneity, the FE approach reveals that the Biocultural Savings (S) pillar is a critical determinant of well-being, effectively capturing the multidimensional reality of the Andean-Amazonian Bioeconomy (AAB) that traditional OLS models often obscure [22,31].

The model reveals that Log Biocultural Savings is a highly significant predictor (*β* = 0.685, *p* < 0.01). Within this log-log specification, the coefficient represents constant elasticity: a 1% increase in the accumulation and reinvestment of biocultural capital corresponds to a 0.685% increase in social well-being. This finding confirms that the preservation of biocultural funds directly translates into human development improvements, such as increased life expectancy. While Log Real per capita GDP remains a strong driver, its contribution must be interpreted alongside the Log Ecological Footprint, which exerts a severe negative pressure on the system (*β*=−31.309, *p* < 0.01). This validates the AAB paradigm’s core thesis: welfare in these territories is not merely a byproduct of income, but a result of the regenerative capacity of biocultural and natural funds [25,32,19].

### 5.1. The primacy of biocultural capital over industrial productivity

The statistical significance of the Biocultural Savings pillar signals a fundamental shift in the development paradigm. Within the AAB framework, well-being is not primarily driven by the speed of resource transformation, but by the maintenance of the biocultural fund [10]. Our findings reveal a “Biocultural Paradox”: while investments in long-term bio-regeneration and biological information [32,33] may appear as costs in immediate consumption-based accounting, they constitute the essential foundation for stable human development [19,20]. This suggests that biocultural capital acts as a complementary driver alongside income, rather than a mere substitute.

### 5.2. Entropic pressure and technological frontiers

The Malmquist Index analysis provides empirical evidence of the Entropic Pressure conceptualized by Georgescu-Roegen [4]. The “technological breathers” observed in countries like Bolivia and Ecuador confirm that biocultural innovation—the application of ancestral knowledge to production—acts as a buffer against thermodynamic limits. However, as noted by Zuniga-Gonzalez [25,33,34], these fluctuations represent a systemic struggle to maintain efficiency within finite biophysical constraints.

Regarding the Indigenous Identity Index (K), the results show a positive but marginally significant effect (*β* = 0.015, *p* < 0.1). This indicates a suggestive positive influence, implying that the impact of indigenous identity on productivity may be mediated by institutional factors or property rights not fully captured in the current dataset, necessitating further empirical exploration [26,35,36].

### 5.3. Transcending traditional metrics: Policy implications

The shift in significance between the preliminary models and the final FE specification confirms that traditional economic indicators are often ‘blind’ to the value of ancestral capital. When metrics are heavily dependent on per capita income, conservation efforts are misclassified as costs [37,38]. Our results demonstrate that once scale disparities are controlled, the accumulation of biocultural capital directly translates into superior outcomes, validating the ‘Vivir Bien’ philosophy as a viable economic strategy [39–41].

To operationalize these findings, public policies must transition toward:

1) Payments for Community Environmental Services (PCES): Providing direct fiscal incentives to indigenous communities for the preservation of biocultural funds [30,36].
2) National Green Accounting Systems: Integrating biocultural assets as recognized productive assets within national statistics to monitor ‘Vivir Bien’ progress as a tangible economic indicator.

From a policy-making perspective, the calculated Malmquist Productivity Index serves as a strategic diagnostic tool for the sampled nations. Regional decision-makers across the Andean and Mesoamerican regions can utilize these indices to identify sectors where technological efficiency is stagnating. For instance, these data justify the reallocation of national budgets toward biotechnology initiatives rooted in ancestral knowledge, where the marginal return on societal well-being is demonstrably higher.

## 6. Conclusions

### 6.1. Recoupling bioeconomy and social well-being

This study demonstrates that the TFP_AAB_ provides greater explanatory power for evaluating development in the Andean-Amazonian context compared to traditional neoclassical TFP models. By integrating biocultural variables and entropic limits, we have validated that social welfare—proxied through life expectancy—is significantly associated with the regenerative capacity of biological and cultural funds. This finding, while specific to the analyzed sample of six countries, suggests that correcting the “blind spots” of traditional models reduces omitted variable bias and allows for a more accurate representation of regional sustainability beyond industrial efficiency.

### 6.2. The biocultural paradox as a development strategy

The identification of the “Biocultural Paradox” constitutes a significant theoretical contribution. This paradox reveals an inverse relationship in accounting: the preservation of natural and ancestral capitals, while appearing as a short-term “cost” in traditional macroeconomic frameworks, constitutes the foundational driver for long-term well-being. Our Fixed Effects estimation confirms this positive correlation, showing that biocultural savings are not merely ethical choices but productive assets. Countries like Bolivia and Ecuador, which have integrated “Vivir Bien” principles, exhibit distinct patterns of resilience. However, these results must be interpreted within the specific scale of each economy, recognizing that larger industrial bases like Mexico’s require careful normalization to isolate biocultural efficiency from industrial throughput.

### 6.3. Overcoming entropic pressure

Through the lens of Georgescu-Roegen’s Entropy Law, we have interpreted the “technological breathers” captured by the Malmquist Index as econometric signals of thermodynamic limits. Persistent entropic pressure, reflected in the substantial negative association between the ecological footprint and longevity (*β*=−31.309, *p* < 0.01), acts as a systemic constraint on human welfare. While this high magnitude reflects a constant elasticity in our log-log specification, it highlights that high-entropy industrial flows can severely offset the gains of GDP growth, requiring a structural shift in how biological information and energy are managed within the territory.

### 6.4. Policy recommendations and implementation mechanisms

To transition toward a high-efficiency bioeconomy, regional governments must institutionalize biocultural savings through the following specific mechanisms:

a) We recommend the implementation of Shadow Pricing and Multi-criteria Dashboards to formally incorporate ancestral practices and ecosystem services as core productive assets within National Green Accounting Systems.
b) Governments should transition from “flow efficiency” (consumption speed) to “fund efficiency” (resource longevity), prioritizing the maintenance of biocultural funds over the maximization of immediate material throughput.
c) To mitigate potential trade-offs, such as reduced industrial tax revenue, we advocate for regional cooperation and the use of international climate financing (e.g., Green Climate Fund) to provide the necessary liquidity to maintain credit stability during the transition to multidimensional welfare metrics.

## Supporting information

S1 File Technical appendix. Detailed mathematical derivations of the expanded production function incorporating Georgescu-Roegen’s thermodynamic principles and the Jach’a Qh’anax framework boundaries.
(PDF)

S2 File Methodology indicators. Complete database matrix (1995–2024) including normalized socio-ecological variables, Min-Max scale distributions, and Human Development Index (HDI) proxy tracking for the studied Mesoamerican and Andean-Amazonian countries.
(DOCX)

S3 File Script R. Replicable R Studio syntax and econometric routine utilized for executing the Fixed Effects (FE) panel data model estimation and the Malmquist Productivity Index operations.
(DOCX)
