# Supplementary material for: The Andean-Amazonian and Mesoamerican Bioeconomy: A new paradigm for productivity and well-being
Source: PLoS One. 2026 Jun 23;21(6):e0345710. doi: 10.1371/journal.pone.0345710 (PMC13289873; doi:10.1371/journal.pone.0345710)
Supplement: S1 File — Detailed mathematical derivations of the expanded production function incorporating Georgescu-Roegen’s thermodynamic principles and the Jach’a Qh’anax framework boundaries. (PDF) [file pone.0345710.s001.pdf]

Technical Appendix: Methodology, Data Operationalization, and Reproducibility Guide

Project: The Andean-Amazonian Bioeconomy: A New Paradigm for Productivity and Well-being

Corresponding Author: Dr. Carlos Alberto Zúniga-González

1. Data Sources and Structure

The primary dataset is a balanced panel covering 6 Latin American countries over the period 1995–2024. All raw data were extracted from the **World Bank Open Data** platform.

- **Dependent Variable (W):** Social Welfare Index, proxied by the **Human Development Index (HDI)**.
- **Independent Variables:** Real GDP per capita (y), Gini Coefficient (G), and Biocultural indicators (Forest Cover, Indigenous Population).

2. Construction of Bioeconomic Proxies

To address the lack of direct market indicators for biocultural assets, the following proxies were constructed:

1. **Biocultural Savings (S):** Calculated as S_{it}= ln (Adjusted Net National Income) - ln(Natural Resource Depletion). This captures the "non-monetary" savings of maintaining biomass.
2. **Social Reciprocity (A):** Proxied through the *Civil Society Participation Index*. High engagement serves as a proxy for communal labor (Minga/Ayni) systems.
3. **Symbolic Capital (K):** Proxied by the *Percentage of Indigenous Population*. It represents the probability of ancestral knowledge retention.

3. Econometric Specification

The model follows the **Sen-Mukhopadhaya Social Welfare Function (SWF)**, linearized through natural logarithms:

$$ln(W_{it}) \ = \ \beta_0 \ + \ \beta_1 \ + \ ln(y_{it}) \ + \ \alpha \ ln \ (1 - \ G_{it} \ + \ \mu_i + \varepsilon_{it}$$

- **Long-Run Estimation:** Performed using Fixed Effects (FE) with **Driscoll-Kraay standard errors** to correct for heteroscedasticity and spatial correlation, as identified in the **Modified Wald Test** ($p < 0.01$) and **Wooldridge Test** ($p < 0.01$).

- **Stationarity & Cointegration:** Unit root tests (**Levin-Lin-Chu** and **Im-Pesaran-Shin**) confirmed that variables are I(1). **Pedroni** and **Kao** tests confirmed a cointegrating relationship ($p < 0.05$).

4. Steps to Reproduce in R

To replicate the results, use the provided datafinal.xlsx and the following logic in R:

1. **Data Cleaning:** Load readxl. Replace missing values using linear interpolation for edge cases ($R^2 > 0.85$).
2. **Panel Setup:** Use pdata.frame(data, index=c("Country", "Year")).
3. **Productivity Analysis:** Calculate the **Malmquist Index** using the nonparaeff or FEAR packages to derive the **TFP-AAB** (Total Factor Productivity of the Andean-Amazonian Bioeconomy).
4. **Regression:** Execute plm(log(HDI) ~ log(gdp) + log(1-Gini), data = p, model = "within").

Appendix: Methodological Step-by-Step for Reproducibility

Step 1: Data Preparation and Transformation

- **Variable Construction:** Use the complement of the Gini coefficient (1 - G) to represent income equality.
- **Logarithmic Transformation:** Apply natural logarithms to GDP per capita and the Social Welfare Index (HDI) to linearize the relationships and interpret results as elasticities.

Step 2: Cross-Sectional Dependence Test

- **Action:** Before testing for stationarity, apply the **Pesaran CD Test**.
- **Justification:** If cross-sectional dependence exists, standard unit root tests may be biased. This justifies the later use of the **Driscoll-Kraay** estimator.

Step 3: Unit Root Testing (TFPAAB / Fisher-ADF)

- **Action:** Conduct the **Fisher-type Augmented Dickey-Fuller (ADF)** unit root test.
- **Procedure:** 1. Test variables in levels: y_t

2. Test variables in first differences: Δy_t.

- **Goal:** Confirm that all variables are integrated of order one, I(1), which is a prerequisite for cointegration.

Step 4: Cointegration Testing (Westerlund / Pedroni)

- **Action:** Apply the **Westerlund (2007)** or **Pedroni** tests for panel cointegration.
- **Goal:** Reject the null hypothesis of "no cointegration" to prove a stable long-term relationship exists between HDI, GDP, and the Gini complement.

Step 5: Long-Term Equation Estimation

- **Model:** Estimate the static long-term equation using **Fixed Effects (FE)**.
- **Formula:**

$$ln(HDI_{it}) = \beta_0 + \beta_1 ln(GDP_{it}) + \beta_2 ln(Equality_{it}) + \mu_i + \varepsilon_{it}$$

- **Validation:** Extract the residuals to ensure they are stationary I(0).

Step 6: Short-Term Dynamics and Error Correction Model (ECM)

- **Action:** Estimate the Error Correction Model following the **Engle-Granger** two-step approach.
- **Formula:**

$$\Delta ln(HDI\}_{it}) = \alpha + \sum \gamma \Delta\ X_{it-1} + \lambda(ECT_{it-1}) + e_{it}$$

- **Key Parameter:** The **Error Correction Term (ECT)** must be negative and statistically significant, representing the speed of adjustment (e.g., 9.64%).

Step 7: Robust Inference (Driscoll-Kraay)

- **Action:** Re-estimate the standard errors using the **Driscoll-Kraay** covariance matrix estimator.
- **Justification:** This ensures that the t-statistics are robust against heteroskedasticity, autocorrelation, and cross-sectional spatial correlation.
