# Supplementary material for: The Andean-Amazonian and Mesoamerican Bioeconomy: A new paradigm for productivity and well-being
Source: PLoS One. 2026 Jun 23;21(6):e0345710. doi: 10.1371/journal.pone.0345710 (PMC13289873; doi:10.1371/journal.pone.0345710)
Supplement: S2 File — Complete database matrix (1995–2024) including normalized socio-ecological variables, Min-Max scale distributions, and Human Development Index (HDI) proxy tracking for the studied Mesoamerican and Andean-Amazonian countries. (DOCX) [file pone.0345710.s002.docx]

**Methodological Proposal for the Construction of Composite Variables of the Model**

The qualitative definitions of its five pillars, extracted from the reference documents, reveal both the complexity and the interconnected nature of its dimensions.

**Community Consumption (C):** Understood as household spending not only on basic goods and services such as food and transportation, but also in areas that foster human development and resilience, such as health, education, and housing.

**Biocultural Savings (S):** This pillar goes beyond monetary savings. It includes collective investment in the preservation of natural and cultural assets, such as participation in cooperatives (for which very little data was found), agroecological projects, and the conservation of local species.

**Reciprocity (A):** Refers to social collaboration and unpaid mutual support. Definitions point to collective work, mutual aid, and participation in community activities, such as the “mingas” in the Nicaraguan context.

**Ecological Balance (E):** This concept operates on a dual scale. At the micro level, it focuses on household environmental conditions (access to water, sanitation, and energy). At the macro level, it encompasses the overall state of the environment, including biodiversity and natural resource management.

**Symbolic Capital (K):** This is the cultural dimension of the model, defined by the preservation of collective identity, the use of the native or local language, and the continuity of cultural and ritual practices.

**2. Methodology for the Construction of Composite Variables**

The construction of composite variables was carried out following a three-step methodology to ensure conceptual consistency and validity.

Identification of Empirical Proxies:

A systematic search was conducted across all available datasets to identify quantitative indicators serving as proxies or representative components of the analyzed concepts. This approach helps overcome the limitation posed by the lack of direct data.

Formulation of the Methodology:

A specific formula is proposed for each variable, detailing the aggregation logic and considerations for data normalization. The goal is to create variables that are not mere sums but reflect the multidimensionality of the conceptual constructs.

Pillar C: Construction of the Community Consumption Variable (C)

3.1 Conceptual Definition and Measurement Challenge

Community consumption is conceptualized as household spending that collectively benefits the household and contributes to the formation of human and social capital. Definitions provided by country (e.g., household expenditure on goods and services such as food, education, health, and transportation for Honduras; expenditure on food, beverages, health, education, and housing for Mexico, etc.) highlight categories of spending that represent investments in long-term stability and development.

3.2 Identification of Proxies and Data Analysis

To operationalize this variable, the dataset CONSUMO DE LOS HOGARES.csv will be used, which provides a detailed breakdown of household expenditures by category. The indicators selected as proxies are:

Health Expenditure

Education Expenditure

Housing, Water, Electricity, Gas, and Other Fuels Expenditure

Food and Non-Alcoholic Beverages Expenditure

Transportation Expenditure

**3.3. Methodology for Creating the Variable**

Community consumption differs from total expenditure in its focus on investment in household resilience. Therefore, a simple sum of all expenditures would not be representative. Instead, a composite variable is proposed that aggregates the expenditure categories with a direct and lasting impact on household quality of life and the development of human capital. Expenditures on health, education, and housing/basic services are the most representative categories of this concept, as they reflect investments in the fundamental well-being of the household. The proposed variable is constructed by summing these per capita expenditures.

Below is the calculation methodology and a demonstrative table using 2021 data for the countries with available information.

The formula for the Community Consumption variable (C) is:

𝐶𝑖𝑡=Health Expenditure𝑖𝑡+Education Expenditure𝑖𝑡+Housing, Water, Electricity Expenditure𝑖𝑡

**Table C.1: Calculation of the Community Consumption Variable (C) (2021)**

| Country | Health Expenditure | Education Expenditure | Housing and Utilities Expenditure | **Variable C ( Communitary Expenditure)** |
| --- | --- | --- | --- | --- |
| Bolivia | 1340.38 | 864.79 | 2374.21 | 4579.38 |
| Ecuador | 1326.91 | 1361.36 | 1855.02 | 4543.29 |
| Guatemala | 1154.22 | 1286.50 | 1648.60 | 4089.32 |
| Honduras | 711.45 | 920.73 | 1060.44 | 2692.62 |
| México | 1246.82 | 5025.92 | 3398.61 | 9671.35 |
| Nicaragua | 1214.59 | 972.09 | 1612.02 | 3798.70 |

**4. Pillar S: Construction of the Biocultural Savings Variable (S)**

4.1. Conceptual Definition and Measurement Challenge

Biocultural Savings represents a form of capital that is not purely monetary. Its definition includes collective investment (participation in cooperatives), community spending or agroecological projects, and participation in rural programs. The main challenge is measuring this qualitative investment in the resilience of ecosystems and culture, given that the variable…

4.2. Identification of Proxies and Data Analysis

Three empirical proxies were identified to capture this multidimensionality:

Agricultural area certified organic (hectares): Measures investment in agroecological practices that promote sustainability.

Plant varieties for which sufficient genetic resources are stored (in quantities): Quantifies the results of investment in plant biodiversity conservation.

Number of local species maintained in the country: Represents the maintenance of local biodiversity, a core component of the “biocultural” concept.

4.3. Methodology for Creating the Variable

These indicators reflect a tangible commitment to long-term sustainability. None of them alone can represent the entirety of the concept, so a composite index is required.

The formula for the Biocultural Savings variable (S) is:

𝑆𝑖𝑡=Composite Index(OrganicAgriculturalArea𝑖𝑡/StoredPlantVarieties𝑖𝑡/MaintainedLocalSpecies𝑖𝑡)

For its calculation, the three components must be normalized to a common scale (from 0 to 1) to allow aggregation. Once normalized, it is proposed to sum them to create the final variable, assigning equal weight to each component.

**Table S.1: Calculation of the Biocultural Savings Variable (S) (2006–2010)**

| Country | Year | Organic Hectáreas | Plants Variety | Locals Species | **Variable S (normalized)** |
| --- | --- | --- | --- | --- | --- |
| Bolivia | 2006 | 0.00 | N/A | 26 | 0.25 |
| Bolivia | 2007 | 0.00 | N/A | 26 | 0.25 |
| Bolivia | 2008 | 0.00 | N/A | 26 | 0.25 |
| Bolivia | 2009 | 0.00 | N/A | 26 | 0.25 |
| Bolivia | 2010 | 92751.9 | 11418 | 26 | 0.96 |
| Ecuador | 2006 | 0.00 | N/A | 27 | 0.26 |
| Ecuador | 2007 | 45900 | N/A | 27 | 0.51 |
| Ecuador | 2008 | 66650 | N/A | 27 | 0.73 |
| Ecuador | 2009 | 64280 | N/A | 27 | 0.70 |
| Ecuador | 2010 | N/A | 25613 | 27 | 0.81 |
| Honduras | 2010 | 15000 | 47 | 21 | 0.53 |
| México | 2010 | 332500 | 59265 | 31 | 0.85 |
| Nicaragua | 2010 | 27100 | 291 | 9 | 0.44 |

**5. Pillar A: Construction of the Reciprocity Variable (A)**

5.1. Conceptual Definition and Measurement Challenge

Reciprocity is the pillar that captures the non-monetary social fabric. Definitions (collective work or unpaid mutual aid, community participation) focus on the value of unpaid work carried out for the benefit of the community. The challenge is to quantify this social work in a representative way.

5.2. Identification of Proxies and Data Analysis

Two key variables were identified: Contributing family workers, male and Contributing family workers, female.

5.3. Methodology for Creating the Variable

Analysis of the data sources reveals an important distinction in the type and magnitude of contributions. Data show that women devote a significantly higher number of weekly hours to unpaid work than men. For example, in Guatemala in 2011, women devoted 52.92 hours while men devoted 23.18 hours. However, the variable “Contributing family workers” in the main database often shows a lower rate for women than for men in some years, suggesting that this variable does not adequately capture caregiving and domestic tasks.

This apparent discrepancy underscores the need for a composite variable that integrates both work intensity (hours) and the proportion of the population contributing. Therefore, the Reciprocity variable should reflect this duality by combining the workload measured in hours with the level of participation.

The formula for the Reciprocity Variable (A) is:

The calculation of variable A can be performed by averaging the data from the percentages of contributing family workers and unpaid working hours by sex. These values will then be normalized to create a composite index that more accurately reflects the concept.

**Table A.1: Calculation of the Reciprocity Variable (A) (2009–2014)**

| Country | Year | Women hours mean | Men hours mean | Family workers % | Familiars worker % | **Variable A (normalized)** |
| --- | --- | --- | --- | --- | --- | --- |
| Guatemala | 2011 | 52.92 | 23.18 | 13.90 | 17.20 | 0.65 |
| Guatemala | 2014 | 34.63 | 10.60 | 8.52 | 13.17 | 0.35 |
| Honduras | 2009 | 30.61 | 14.09 | 8.21 | 9.30 | 0.28 |
| México | 2009 | 44.37 | 16.35 | 4.67 | 9.52 | 0.44 |
| México | 2014 | 42.14 | 15.36 | 4.00 | 8.03 | 0.40 |

**6. Pillar E: Construction of the Ecological Equilibrium Variable (E)**

**6.1. Conceptual Definition and Measurement Challenges**

Ecological Equilibrium encompasses both environmental conditions at the household level and ecosystem health on a broader scale. Country-specific definitions confirm this by including access to water, sanitation, and energy alongside biodiversity and environmental conservation.

**6.2. Identification of Proxies and Data Analysis**

To overcome the lack of direct data, indicators from two levels of analysis are combined:

**Micro Dimension (Household):**

**People using safely managed drinking water services (% of population).**

**Percentage of the population using at least basic sanitation services.**

**Access to electricity (% of population).**

**Macro Dimension (Ecosystem):**

**Area of wetlands under the Ramsar Convention (hectares).**

**Greenhouse Gas (GHG) Emissions per GDP.**

**Proportion of forest area under a long-term management plan.**

**6.3. Variable Construction Methodology**

Ecological Equilibrium is a resilience metric manifested in both household welfare and environmental health. The construction of this variable requires combining indicators from both dimensions, as relying on a single scale would result in an incomplete perspective. Access to services such as water and sanitation reflects the household's immediate environmental quality, while GHG emissions, wetland area, and forest management measure sustainability at the regional or national level.

A composite index is proposed, consisting of two normalized and weighted sub-indices:

Ecosystem Quality Sub-index: Normalization of "Area of wetlands" and "Proportion of forest area," and the inversion and normalization of "Greenhouse Gas (GHG) Emissions per GDP" (since higher emissions imply lower equilibrium).

Household Conditions Sub-index: Normalization of "People using safely managed drinking water services," "Percentage of the population using at least basic sanitation services," and "Access to electricity."

.Tabla E.1: Cálculo de la Variable Equilibrio Ecológico (E) (2015)

| Country | Year | Segure water% | Basic sanitation% | Hectáreas Humedales | EmissionsGEI/GDP | Forest management% | **Variable E (normalized)** |
| --- | --- | --- | --- | --- | --- | --- | --- |
| Bolivia | 2015 | N/D | 57 | 14842405 | 606.14 | 22.14 | 0.44 |
| Ecuador | 2015 | N/D | 65 | 290815 | 416.86 | 44.29 | 0.58 |
| Guatemala | 2015 | N/D | 67 | 628592 | 264.62 | 0.16 | 0.35 |
| Honduras | 2015 | N/D | 78 | 270224 | 502.30 | N/A | 0.33 |
| México | 2015 | N/D | 88 | 8657056.54 | 402.46 | 12.52 | 0.60 |
| Nicaragua | 2015 | N/D | 72 | 406852 | 414.43 | 0.40 | 0.40 |

**7. Pillar K: Construction of the Symbolic Capital Variable (K)**

7.1. Conceptual Definition and Measurement Challenge

Symbolic Capital is associated with cultural identity, ritual practices, and the use of the native language. Country definitions refer to Indigenous self-identification, cultural participation, and local language use. It is an intangible concept, and the main challenge is finding a demographic proxy that adequately represents it.

7.2. Identification of Proxies and Data Analysis

The document población indigena.csv provides the number of people who self-identify as Indigenous or Afro-descendant in various countries and decades. This demographic presence is the strongest empirical indicator of the existence of Symbolic Capital. The percentage of the total population belonging to these groups is a useful metric to measure the reach of this capital within a society.

7.3. Methodology for Creating the Variable

It is acknowledged that the percentage of Indigenous or Afro-descendant population is a proxy, not a direct measure of cultural vitality. The presence of these demographic groups is a necessary condition for this capital to exist, but it does not quantify the intensity of cultural transmission, language use, or ritual participation. Despite this limitation, this is the best possible approximation with the available data and provides a solid basis for analysis.

The formula for the Symbolic Capital Variable (K) is:

For calculation, the Indigenous and Afro-descendant populations of each country are summed and divided by the total population of the corresponding year.

Table K.1: Calculation of the Symbolic Capital Variable (K) (2010s Decade)

| Country | Decade | Indigenous Population(Miles) | Afrodescendiente population(Miles) | Total Populationl (2010) (Miles) | **Variable K (%)** |  |  |
| --- | --- | --- | --- | --- | --- | --- | --- |
| Bolivia | 2010 | 4176647 | 23330 | 10182345 | 41.24% |  |  |
| Ecuador | 2010 | 1018176 | 1041559 | 15076695 | 13.66% |  |  |
| Guatemala | 2010 | 6471670 | 47176 | 14500112 | 44.96% |  |  |
| Honduras | 2010 | 601824 | 115794 | 8365878 | 8.58% |  |  |
| México | 2010 | 15708995 | N/A | 113623895 | 13.83% |  |  |
| Note: Total population data come from BASE DE DATOS.csv for 2010, and data on Indigenous and Afro-descendant populations come from población indigena.csv. Total population is used for the decade due to the lack of exact annual values in the source for the decade. | | | | | |  |  |

**8. Discussion, Limitations, and Recommendations8.1. Integration of the New Variables**

8.1 The five composite variables (C, S, A, E, K) have been formulated with a focus on conceptual validity and the optimized utilization of available data.

They represent a robust foundation for any quantitative analysis of the Model. With these variables, it is possible to conduct a multivariate analysis to study their interconnection and, fundamentally, their relationship with the Life Satisfaction Index.

**8.2. Limitations of the Approach**

Despite the methodological rigor, the composite variables are subject to several limitations, mainly related to data availability.

Data Availability and Overlap: Data for each variable come from different sources and have limited temporal and geographic coverage. For example, detailed consumption data (C) are available only for 2017 and 2021, whereas data on unpaid work (A) cover sporadic years and countries.

Nature of the Proxies: It must be acknowledged that the S and K variables, in particular, are proxies and do not capture the full scope of their concepts. The S variable does not distinguish the origin of agroecological investment, while the K variable does not measure cultural vitality or practice, only demographic presence.

Absence of Key Variables: The lack of data for indicators such as local ecosystem health and biodiversity in the main database is a significant limitation for constructing a more robust E variable. Nevertheless, efforts will continue to locate data to strengthen it.
