# Supplementary material for: The Andean-Amazonian and Mesoamerican Bioeconomy: A new paradigm for productivity and well-being
Source: PLoS One. 2026 Jun 23;21(6):e0345710. doi: 10.1371/journal.pone.0345710 (PMC13289873; doi:10.1371/journal.pone.0345710)
Supplement: S3 File — Replicable R Studio syntax and econometric routine utilized for executing the Fixed Effects (FE) panel data model estimation and the Malmquist Productivity Index operations. (DOCX) [file pone.0345710.s003.docx]

Script Table 3

# 1. Load necessary libraries

library(readxl)

library(dplyr)

library(deaR)

library(zoo)

library(tidyr)

# 2. Upload your data

Dataforreproductibility <- read_excel("D:/Publications 2026/Plos One/Dataforreproductibility.xlsx")

# 3. Cleaning and Selection of Variables (Table 1)

data_clean <- Dataforreproductibility %>%

select(Country = 1,

Year = 2,

biocultural_savings_S = 28,

Indigenous_population_K = 20,

Forest_cover_biodiversity_E = 22,

Political_social_participation_A = 18,

household_consumption_C = 27) %>%

# Ensure panel order: Country then Year

arrange(Country, Year) %>%

group_by(Country) %>%

# Handling Missing Elements: Linear Interpolation

mutate(across(c(biocultural_savings_S, Indigenous_population_K,

Forest_cover_biodiversity_E, Political_social_participation_A,

household_consumption_C),

~na.approx(., na.rm = FALSE))) %>%

ungroup()

# 4. Robust Normalization

# Scale to [0.001, 1] to avoid zeros and ensure numerical stability

normalize_robust <- function(x) {

if(all(is.na(x))) return(x)

min_x <- min(x, na.rm = TRUE)

max_x <- max(x, na.rm = TRUE)

if(max_x == min_x) return(rep(0.5, length(x))) # Si no hay variación, poner valor medio

# Normalized Min-Max between 0.001 and 1

norm_x <- 0.001 + (x - min_x) * (1 - 0.001) / (max_x - min_x)

return(norm_x)

}

# Apply standardization

data_norm <- data_clean %>%

mutate(across(c(biocultural_savings_S,

Indigenous_population_K,

Forest_cover_biodiversity_E,

Political_social_participation_A,

household_consumption_C),

normalize_robust))

# 5. --- Prepare Malmquist object ---

inputs_vars <- c("biocultural_savings_S", "Indigenous_population_K",

"Forest_cover_biodiversity_E", "Political_social_participation_A")

output_vars <- "household_consumption_C"

anios <- unique(data_norm$Year)

data_malm <- make_malmquist(data = as.data.frame(data_norm),

percol = "Year",

arrangement = "vertical",

nper = length(anios),

inputs = inputs_vars,

outputs = output_vars)

# 6. Calculate the Malmquist Index

# Orientation: Output-Oriented (oo), Variable Returns (vrs)

results_malmquist <- malmquist_index(data_malm,

orientation = "oo",

rts = "vrs")

# 7. Generate Summary

summary_table3 <- summary(results_malmquist)

print(summary_table3Results)

print(summary_table3means_by_dmu)

# 7. Generate Summary

> summary_table3 <- summary(results_malmquist)

> print(summary_table3Results)

Period DMU mi tc pech sech

1 1996 Bolivia 1.0420826 0.9011999 2.0620130 0.5607763

2 1996 Ecuador 0.9360912 0.5645297 1.0000000 1.6581788

3 1996 Guatemala 0.7034549 0.7034549 1.0000000 1.0000000

4 1996 Honduras 0.8395847 0.9186709 0.9371676 0.9751856

5 1996 México 1.1247973 1.1247973 1.0000000 1.0000000

6 1996 Nicaragua 1.0631318 0.8972693 1.2150270 0.9751655

7 1997 Bolivia 1.0519247 1.1655668 1.2730921 0.7089044

8 1997 Ecuador 0.9742712 1.1746977 1.0000000 0.8293803

9 1997 Guatemala 1.1360231 1.1360231 1.0000000 1.0000000

10 1997 Honduras 1.1095281 1.1192195 5.9919468 0.1654456

11 1997 México 1.3694521 1.3694521 1.0000000 1.0000000

12 1997 Nicaragua 1.2005370 1.1704475 5.5103349 0.1861425

13 1998 Bolivia 1.1007618 1.1242524 0.3992175 2.4525619

14 1998 Ecuador 0.9491015 1.0626395 1.0000000 0.8931548

15 1998 Guatemala 1.1261412 1.1261412 1.0000000 1.0000000

16 1998 Honduras 1.2250576 1.0781596 1.0000000 1.1362489

17 1998 México 1.2752317 1.2752317 1.0000000 1.0000000

18 1998 Nicaragua 0.9754440 1.1197990 0.1582383 5.5049171

19 1999 Bolivia 1.0454469 1.0798437 1.1976687 0.8083592

20 1999 Ecuador 0.6718237 1.0760097 1.0000000 0.6243658

21 1999 Guatemala 1.0879889 1.0879889 1.0000000 1.0000000

22 1999 Honduras 1.1613928 1.0910547 0.1975351 5.3887536

23 1999 México 1.0511578 1.0511578 1.0000000 1.0000000

24 1999 Nicaragua 1.1595909 1.0814200 1.0940163 0.9801366

25 2000 Bolivia 1.1908782 1.3171566 0.7125767 1.2688151

26 2000 Ecuador 0.8359431 1.5183131 0.1904205 2.8913569

27 2000 Guatemala 1.4874888 1.4874888 1.0000000 1.0000000

28 2000 Honduras 1.5361971 1.6508241 5.0623915 0.1838190

29 2000 México 5.2696147 5.2696147 1.0000000 1.0000000

30 2000 Nicaragua 1.1031365 1.2825977 0.8423647 1.0210304

31 2001 Bolivia 1.0006457 1.0336953 1.0007064 0.9673443

32 2001 Ecuador 1.0190732 1.0850427 0.9515695 0.9870019

33 2001 Guatemala 1.1180352 1.1180352 1.0000000 1.0000000

34 2001 Honduras 1.1603445 0.9904584 1.0000000 1.1715227

35 2001 México 0.7844148 0.7844148 1.0000000 1.0000000

36 2001 Nicaragua 0.9807380 1.0280619 0.9681517 0.9853496

37 2002 Bolivia 0.8673663 0.9375093 1.0600102 0.8728044

38 2002 Ecuador 0.8367124 0.8607944 0.9786331 0.9932461

39 2002 Guatemala 0.8069821 0.8069821 1.0000000 1.0000000

40 2002 Honduras 0.6285991 0.6806101 1.0000000 0.9235819

41 2002 México 0.6548984 0.6548984 1.0000000 1.0000000

42 2002 Nicaragua 0.9039851 0.9745851 7.0830642 0.1309545

43 2003 Bolivia 1.0111620 1.0629841 0.8225003 1.1565327

44 2003 Ecuador 1.1850164 1.1682032 1.0266416 0.9880686

45 2003 Guatemala 1.2583115 1.2583115 1.0000000 1.0000000

46 2003 Honduras 1.0361158 1.0006984 1.0000000 1.0353927

47 2003 México 1.1574200 1.1574200 1.0000000 1.0000000

48 2003 Nicaragua 0.9737137 1.0443593 0.1217269 7.6593999

49 2004 Bolivia 0.9839439 0.9493523 2.7053514 0.3831063

50 2004 Ecuador 0.9738033 0.9452951 1.0276683 1.0024227

51 2004 Guatemala 0.9141106 0.9141106 1.0000000 1.0000000

52 2004 Honduras 0.9038729 0.9711237 0.2121514 4.3871956

53 2004 México 1.1099815 1.1099815 1.0000000 1.0000000

54 2004 Nicaragua 1.1427624 0.9508489 8.2151098 0.1462955

55 2005 Bolivia 0.8678668 0.9633737 0.3370028 2.6731592

56 2005 Ecuador 0.8709177 0.8577393 0.9859040 1.0298812

57 2005 Guatemala 0.8549044 0.8549044 1.0000000 1.0000000

58 2005 Honduras 0.9571360 1.0306408 0.8288561 1.1204364

59 2005 México 0.6292433 0.6292433 1.0000000 1.0000000

60 2005 Nicaragua 0.8646051 1.0029046 0.1246507 6.9161361

61 2006 Bolivia 1.0437030 1.0440359 1.0023422 0.9973452

62 2006 Ecuador 1.0523544 1.0859383 0.9696914 0.9993631

63 2006 Guatemala 1.1441635 1.1441635 1.0000000 1.0000000

64 2006 Honduras 1.0741338 1.0095466 5.6868930 0.1870927

65 2006 México 1.2686764 1.2686764 1.0000000 1.0000000

66 2006 Nicaragua 1.1338297 1.0259536 8.0224192 0.1377573

67 2007 Bolivia 0.8913200 0.9298652 0.9740369 0.9840978

68 2007 Ecuador 0.7876067 0.8969620 0.9059126 0.9692796

69 2007 Guatemala 0.8868946 0.8868946 1.0000000 1.0000000

70 2007 Honduras 0.8838165 0.9603373 0.1732102 5.3133067

71 2007 México 0.6329068 0.6329068 1.0000000 1.0000000

72 2007 Nicaragua 1.0967246 1.0165052 1.0000000 1.0789169

73 2008 Bolivia 1.0124240 1.0769409 0.9403645 0.9997107

74 2008 Ecuador 1.1609629 1.1732810 1.0589912 0.9343809

75 2008 Guatemala 1.2657493 1.2657493 1.0000000 1.0000000

76 2008 Honduras 1.1117593 1.0175894 1.0938007 0.9988493

77 2008 México 1.0638496 1.0638496 1.0000000 1.0000000

78 2008 Nicaragua 1.0895889 1.0149371 1.0000000 1.0735531

79 2009 Bolivia 0.9481119 0.9519677 0.9817083 1.0145068

80 2009 Ecuador 0.9612392 0.9581926 5.8278350 0.1721359

81 2009 Guatemala 0.9527707 0.9527707 1.0000000 1.0000000

82 2009 Honduras 1.0592329 0.9493013 5.2782303 0.2113971

83 2009 México 1.2289207 1.2289207 1.0000000 1.0000000

84 2009 Nicaragua 0.7841233 0.9624459 1.0000000 0.8147194

85 2010 Bolivia 4.3755923 4.5893995 0.9490609 1.0045855

86 2010 Ecuador 3.4505479 3.2565983 1.0000000 1.0595559

87 2010 Guatemala 3.0223340 3.0223340 1.0000000 1.0000000

88 2010 Honduras 5.8977043 5.9752362 0.2626723 3.7576270

89 2010 México 17.1157585 17.1157585 1.0000000 1.0000000

90 2010 Nicaragua 5.7406997 5.0240006 1.0000000 1.1426550

91 2011 Bolivia 1.1110569 1.0355402 2.4545621 0.4371146

92 2011 Ecuador 1.0863535 1.0762382 1.0000000 1.0093988

93 2011 Guatemala 1.1803960 1.1803960 1.0000000 1.0000000

94 2011 Honduras 1.0488658 1.0266759 3.8070252 0.2683495

95 2011 México 0.8337811 0.8337811 1.0000000 1.0000000

96 2011 Nicaragua 1.1272500 1.0286855 1.0000000 1.0958160

97 2012 Bolivia 0.4061588 0.4238352 0.4207230 2.2777321

98 2012 Ecuador 0.6247593 0.6104718 1.0000000 1.0234041

99 2012 Guatemala 0.4411156 0.4411156 1.0000000 1.0000000

100 2012 Honduras 0.3214447 0.3169874 0.4393594 2.3080451

101 2012 México 0.1617378 0.1617378 1.0000000 1.0000000

102 2012 Nicaragua 0.4967143 0.4700029 1.0000000 1.0568325

103 2013 Bolivia 1.0807706 1.0347707 1.0407306 1.0035779

104 2013 Ecuador 1.1157645 1.0617124 1.0000000 1.0509103

105 2013 Guatemala 0.9972733 0.9972733 1.0000000 1.0000000

106 2013 Honduras 1.1695438 1.0033352 2.2760409 0.5121420

107 2013 México 1.2576782 1.2576782 1.0000000 1.0000000

108 2013 Nicaragua 1.0223305 1.0267135 1.0000000 0.9957310

109 2014 Bolivia 0.8299096 1.0202522 0.8234880 0.9877930

110 2014 Ecuador 0.9983653 1.0514346 1.0000000 0.9495268

111 2014 Guatemala 1.1055522 1.1055522 1.0000000 1.0000000

112 2014 Honduras 0.9934240 1.0073684 1.0000000 0.9861577

113 2014 México 0.8589579 0.8589579 1.0000000 1.0000000

114 2014 Nicaragua 1.0037424 1.0159644 1.0000000 0.9879700

115 2015 Bolivia 6.3705775 4.4243378 7.8394227 0.1836735

116 2015 Ecuador 3.8426690 2.4596545 1.0000000 1.5622800

117 2015 Guatemala 2.7283989 2.7283989 1.0000000 1.0000000

118 2015 Honduras 3.2501968 3.2255371 0.2673476 3.7690457

119 2015 México 9.5464435 9.5464435 1.0000000 1.0000000

120 2015 Nicaragua 3.0792939 2.8662176 1.0000000 1.0743406

121 2016 Bolivia 0.9115071 0.9886706 0.4119486 2.2380276

122 2016 Ecuador 0.6078283 1.0179951 1.0000000 0.5970837

123 2016 Guatemala 1.0809256 1.0809256 1.0000000 1.0000000

124 2016 Honduras 1.0041448 0.9957913 3.7404494 0.2695903

125 2016 México 0.8337045 0.8337045 1.0000000 1.0000000

126 2016 Nicaragua 0.9186279 0.9921680 1.0000000 0.9258793

127 2017 Bolivia 0.3656276 0.3389573 0.4784726 2.2544306

128 2017 Ecuador 0.5374918 0.4658795 1.0000000 1.1537142

129 2017 Guatemala 0.3590034 0.3590034 1.0000000 1.0000000

130 2017 Honduras 0.2776236 0.2740538 0.2835053 3.5732178

131 2017 México 0.1573889 0.1573889 1.0000000 1.0000000

132 2017 Nicaragua 0.9538072 0.9298471 1.0000000 1.0257678

133 2018 Bolivia 0.9785677 1.0190657 0.9059128 1.0599914

134 2018 Ecuador 0.7792892 1.1143035 0.1366153 5.1191261

135 2018 Guatemala 1.2651217 1.2651217 1.0000000 1.0000000

136 2018 Honduras 1.0560866 1.0029428 0.9542622 1.1034576

137 2018 México 0.9092329 0.9092329 1.0000000 1.0000000

138 2018 Nicaragua 1.0192348 0.9896001 1.0000000 1.0299462

139 2019 Bolivia 1.0143888 1.0166673 5.6003286 0.1781608

140 2019 Ecuador 0.9773652 0.9368508 7.3198241 0.1425233

141 2019 Guatemala 1.0232194 1.0232194 1.0000000 1.0000000

142 2019 Honduras 1.0784324 1.0092688 1.0676460 1.0008266

143 2019 México 1.4294680 1.4294680 1.0000000 1.0000000

144 2019 Nicaragua 0.9424034 0.9941695 1.0000000 0.9479303

145 2020 Bolivia 0.9802335 1.1013011 0.1845760 4.8222342

146 2020 Ecuador 1.5055878 1.6888417 0.1191148 7.4843070

147 2020 Guatemala 2.1247888 2.1247888 1.0000000 1.0000000

148 2020 Honduras 1.0931117 1.0780932 1.0030769 1.0108205

149 2020 México 2.5867106 2.5867106 1.0000000 1.0000000

150 2020 Nicaragua 0.9176287 0.9362196 1.0000000 0.9801426

151 2021 Bolivia 0.9721385 1.1026730 0.7349598 1.1995486

152 2021 Ecuador 1.0930082 1.1284854 0.9687774 0.9997778

153 2021 Guatemala 3.2729967 3.2729967 1.0000000 1.0000000

154 2021 Honduras 1.5479516 1.0694790 3.4515135 0.4193489

155 2021 México 1.0228731 1.0228731 1.0000000 1.0000000

156 2021 Nicaragua 1.3664538 1.0599740 1.0000000 1.2891390

157 2022 Bolivia 0.8010716 0.8594015 0.9708203 0.9601441

158 2022 Ecuador 0.5605380 0.5758611 8.6658357 0.1123251

159 2022 Guatemala 0.4970629 0.4970629 1.0000000 1.0000000

160 2022 Honduras 0.4765619 0.5472108 1.0000000 0.8708927

161 2022 México 0.7858397 0.7858397 1.0000000 1.0000000

162 2022 Nicaragua 3.0351186 1.0877397 1.0000000 2.7902988

163 2023 Bolivia 1.0517903 1.1315312 0.9606107 0.9676431

164 2023 Ecuador 1.1549276 1.4500426 0.1034244 7.7010682

165 2023 Guatemala 1.2587443 1.2587443 1.0000000 1.0000000

166 2023 Honduras 1.0279007 1.2004873 0.3473666 2.4649350

[ reached 'max' / getOption("max.print") -- omitted 8 rows ]

> print(summary_table3means_by_dmu)

DMU mi tc pech sech

1 Bolivia 1.0429509 1.057581 1.050923 0.9383816

2 Ecuador 0.9970214 1.044230 1.000000 0.9547905

3 Guatemala 1.0882468 1.088247 1.000000 1.0000000

4 Honduras 1.0306030 1.010941 1.019647 0.9998066

5 México 1.1174231 1.117423 1.000000 1.0000000

6 Nicaragua 1.2218767 1.144334 1.067762 1.0000000

**R Code for Figure 1 (Updated Label)**

library(ggplot2)

library(dplyr)

# 1. Prepare the data

# Assuming 'summary_table3$Results' is your data frame

df_plot <- summary_table3$Results

# Ensure Period is numeric

df_plot Period <- as.numeric(as.character(df_plot$Period))

# 2. Generate the trend graph (Figure 1)

figura1 <- ggplot(df_plot, aes(x = Period, y = mi, color = DMU, group = DMU)) +

geom_line(size = 1) + # Lines for trend

geom_point(size = 2) + # Points for each year

geom_hline(yintercept = 1, linetype = "dashed", color = "red") + # Equilibrium line

theme_minimal() + # Clean theme

labs(title = "Trends in Bioeconomic Productivity (TFP-AAB)",

subtitle = "Annual change by country (1996-2023)",

x = "Year",

y = "TFP-AAB", # Updated label

color = "Country") +

theme(legend.position = "bottom",

plot.title = element_text(face = "bold"))

# 3. Display the graph

print(figura1)

# 4. (Optional) Save the graph

# ggsave("Figure1_Trends_TFP_AAB.png", figura1, width = 10, height = 6, dpi = 300)

R code for Table 4

# ==============================================================================

# TABLE 4 - STEP 1: DEFINING PROXY FOR WELFARE FUNCTION (WITHOUT GINI)

# ==============================================================================

# 1. We will use the adjusted net national income as the income proxy

data_p$Welfare_Proxy <- data_p$`Adjusted_.net_national_income_per_capita_.constant_2015_US..`

# ==============================================================================

# TABLE 4 - STEP 2: RUN PRELIMINARY REGRESSION

# ==============================================================================

# Using the columns found in colnames(data_p)

model_table4 <- lm(Welfare_Proxy ~ TFP_AAB + `biocultural_savings_.S.` + `Life_expectancy_at_birth`,

data = data_p)

# Print results

summary(model_table4)

R code for Table 5 # ==============================================================================

# TABLE 5 - FORCED RENAMING STRATEGY

# ==============================================================================

# 1. AGGRESSIVELY CLEAN THE COLUMN NAME

# Search for the column resembling "Indigenous_population_[K]" and assign a clean name

colnames(data_p)[grep("Indigenous_population", colnames(data_p))] <- "Indigenous_population_K"

# 2. RUN THE MODEL USING THE NEW CLEAN NAME

model_table5 <- plm(Life_Expectancy ~ Log_Biocultural_Savings +

Indigenous_population_K + Log_Real_GDP +

Log_Ecological_Footprint,

data = data_p,

model = "within")

# 3. PRINT TABLE 5

stargazer(model_table5, type = "text",

title = "Fixed Effects Regression Results (Proxy: Life Expectancy / Well-being)",

covariate.labels = c("Log Biocultural Savings",

"Indigenous Population (Index K)",

"Log Real per capita GDP",

"Log Ecological Footprint (CO2)"),

dep.var.labels = "Life Expectancy")
