# Supplementary material for: The Andean-Amazonian and Mesoamerican Bioeconomy: A new paradigm for productivity and well-being
Source: PLoS One. 2026 Jun 23;21(6):e0345710. doi: 10.1371/journal.pone.0345710 (PMC13289873; doi:10.1371/journal.pone.0345710)
Supplement: Attachment — Submitted filename: Peer Review_PLOS One.pdf [file pone.0345710.s004.pdf]

The Andean-Amazonian Bioeconomy: A New Paradigm for Productivity and Well-being
--Manuscript Draft—

As a peer reviewer for *PLOS ONE*, I’ve evaluated the manuscript based on the journal’s standards for scientific rigor, clarity, and methodological transparency.

Abstract

Line	Feedback & Suggestions
24	You mention "Fixed Effects (FE) panel data model." To be more "PLOS-like," briefly mention the dependent variable used to represent "social welfare" (e.g., Human Development Index, Life Expectancy, or a custom index).
28	While you call it a "Biocultural Paradox," your findings actually seem to *resolve* the paradox by showing a positive correlation. You might consider phrasing it as "Addressing the Biocultural Paradox...
29	You mention "Biocultural Savings." In the context of the Andean-Amazonian region, is this synonymous with "Genuine Savings" or "Adjusted Net Savings," or is it a proprietary metric? A quick clarification in the methodology section of the full paper will be vital.
28-31	“Our findings reveal a 'Biocultural Paradox': while traditional income-based metrics may view conservation as a cost, the refined model demonstrates that Biocultural Savings is a positive and highly significant predictor of social well-being ($\beta = 0.685$, $p < 0.01$), alongside a positive impact from the indigenous population index ($\beta = 0.015$, $p < 0.1$).”<br><br>Sentence is too long and dense, can you break into two sentences and briefly clarify the term?
31	How do you feel about the $p < 0.1$ significance level for the indigenous index. Do you think the data supports a stronger causal claim in the full manuscript?
32	“Biocultural fund” is unclear (new term not introduced earlier) so should ensure consistency with “biocultural savings”

Introduction

Line	Feedback on Shortcomings & Deficiencies
39	Vagueness: "Civilizational crisis" is a heavy, somewhat dramatic term for a scientific paper. Be more specific about the "ecological and social imbalances" (e.g., climate change, biodiversity loss, or wealth inequality).
41	Redundancy: The phrase "disconnected from its biophysical underpinnings" and "demystified by Georgescu-Roegen" covers the same ground. You can merge these to improve flow.

43	Technical transition: The explanation of entropy is standard. However, the connection between "high-entropy waste" and the "Andean-Amazonian Bioeconomy" needs a stronger bridge. *How* exactly do AAB reverse or mitigate this flow?
47	Conceptual density: You introduce "cosmic-territorial balance," "community reciprocity," and "Buen Vivir" all at once. For a global audience, these terms need a brief, one-sentence operational definition within the text.
48-49	Repetition: This section repeats the reference to Georgescu-Roegen and the Law of Entropy mentioned in lines 48-49. This is a significant deficiency; you should not introduce the same theorist and concept twice in the same paragraph.
50	Structural placement: The mention of "Menéndez Gámiz et al." is useful, but the "theoretical gap" regarding TFP (Total Factor Productivity) feels like it should have been introduced earlier when discussing the "prevailing economic approach."
59	Contribution clarity: You state the contribution is "twofold," but the description is slightly wordy. Ensure the distinction between the "theoretical framework" and "empirical findings" is sharp.
62	Redundancy alert: This sentence ("In doing so, this work responds to the need...") is nearly identical to the final sentence of the text. Delete one of them.
66	Methodological jump: The transition to Amartya Sen's "capability approach" is sudden. You need a clearer sentence explaining *why* Sen's approach is the appropriate lens for measuring the "Well-being" mentioned in your title.
69	Technical detail: Moving from high-level philosophy (*Buen Vivir*) to "Fixed Effects (FE) models" is a jarring shift. Use a transitional phrase like "To empirically validate these theoretical propositions..."

<u>Literature Review</u>

**Line / Sentence**	**Feedback on Shortcomings & Deficiencies**
Line 73-75: "The literature reveals... social imperatives [12, 13]."	Lack of breadth: While identifying the three perspectives (resource, tech, ecology) is standard, the sentence fails to explain *why* they have failed to integrate social imperatives. It misses a chance to cite the specific tensions between "Green Growth" and "Degrowth" literature.
Line 75-77: "Within this context... modern biophysical science [12]."	Definitional circularity: The text uses reference [12] to support both the problem and the solution. This suggests a narrow citation base. It also doesn't define what "modern biophysical science" specifically entails in this context (e.g., Thermodynamics? Ecology?).

Line 78-79: "The AAB is fundamentally... Law of Entropy to economic processes."	Repetition from introduction: This information was already heavily emphasized in the Introduction. In a Literature Review, you should move beyond the *fact* of entropy and discuss *how* other authors have attempted (and perhaps failed) to apply it to bioeconomy.
Line 79-82: "Unlike neoclassical... inexhaustible flows, the AAB framework recognizes the biophysical limits..."	Over-simplification: Attacking the Cobb-Douglas model is a common trope in ecological economics. To be scientifically rigorous for *PLOS ONE*, you should acknowledge more recent neoclassical attempts to include "Natural Capital" (e.g., Dasgupta Review) and explain why AAB is superior.
Line 82-83: "As noted by Quiroga Canavari [14]... degradation of energy and matter."	Lack of "How": This is a purely theoretical statement. The deficiency here is the lack of a "bridge" explaining how this transition is measured or observed in the Andean-Amazonian context.
Line 83-86: "To operate this, the Jach'a Qh'anax model provides... [11, 15]."	Conceptual Opaque-ness: You introduce a highly specific, non-Western term ("Jach'a Qh'anax") without a brief parenthetical definition or translation. For a global audience, this creates a barrier to understanding.
Line 87-91: "The AAB framework operationalizes... redefine the inputs of the production function:"	Premature conclusion: This sentence shifts from reviewing literature to describing your own model's mechanics. This belongs in the Methodology or Theoretical Framework section rather than the Literature Review.
Line 87-91: "(i) Bioculture... (ii) Bio territoriality..."	Missing pillar: The text mentions "three core pillars" but only lists two. This is a significant structural deficiency. Furthermore, describing these as "redefined inputs" is a claim that needs to be supported by showing how others have tried and failed to quantify "ancestral knowledge."

<u>Materials and Methods</u>

3.1. Construction of Composite Indicators and Reproducibility

Line / Sentence	Feedback on Shortcomings & Deficiencies
Line 94-96: "The empirical analysis... (Bolivia, Colombia, Ecuador, Peru, Mexico, and Nicaragua) for the period 1995 2024 [37]."	Geographic inconsistency: The title and theory focus on the Andean-Amazonian region, yet the sample includes Mexico and Nicaragua. You must justify why these non-Amazonian countries are included in a paradigm specifically named after the Andes and Amazon.
Line 97-99: "Data were harmonized from... (RICYT)."	Source specificity: While you list the organizations, you do not name the specific databases or indicators used (e.g.,

	"World Development Indicators" vs "Health Nutrition and Population statistics"). This hinders reproducibility.
Line 98-101: "To ensure comparability... linear interpolation to maintain the continuity of the entropic flow analysis."	Methodological risk: Linear interpolation is a "weak" method for handling missing values in long-term panel data (1995–2024), especially for volatile economic or ecological indicators. You should justify why more robust methods (like Multiple Imputation or Kalman Filters) were not used.
Line 102-104: "To ensure reproducibility... detailed in Table 1."	Redundancy: This sentence essentially repeats the information provided in the first two sentences of the section.
Line 104-105: "Due to the different units... all variables were normalized."	Repetition: This sentence appears almost identically later in the text. This is a significant drafting error.
Line 105-111: "To ensure numerical robustness... scaling all bioeconomic variables to a range of [0.001, 1]... eliminates absolute zeros..."	Clarification needed: While the math is sound for DEA (Data Envelopment Analysis), you must explain *why* 0.001 was chosen as the floor. Does this specific epsilon value affect the sensitivity of the efficiency scores?
Line 109-111: "Additionally, to manage constant time series... a constant mean value of 0.5 was established..."	Statistical bias: Assigning a value of 0.5 to series with no variance is highly unconventional and potentially biased. If a variable doesn't change over time, it provides no information for a Fixed Effects model. You must justify why these variables aren't simply dropped.
Line 111-115: "Finally, the integrity of the data panel... under an output-oriented model with variable returns to scale."	Software-Specific Language: The phrase arrange (Country, Year) looks like R code snippet. Avoid using coding syntax in the prose; instead, describe the logic. Also, you mention an "output-oriented model" here for the first time—this belongs in a sub-section defining the DEA model itself.
Line 115-118: "Due to the different units... all variables were normalized using the Min-Max method... (Eq. 1):"	Direct contradiction: This sentence contradicts the earlier statement. Earlier, you said you scaled to [0.001, 1], but here you say [0, 1]. You must choose one and be consistent. Using $[0, 1]$ will cause the "infinity" errors you previously claimed to avoid.

3.2. Data management and biocultural savings formulation

**Line / Sentence**	**Feedback on Shortcomings & Deficiencies**
Line 121-123: "Missing data points... linear interpolation and extrapolation..."	Technical weakness: Extrapolation is highly risky for a 30-year span (1995–2024), especially if the missing data occurs at the beginning or end of the series. You must specify the percentage of missing data; if it exceeds 10–15%, the "robustness" claim is scientifically questionable.

Line 123-125: "The final Biocultural Savings (S) represents the net national income adjusted by these socio-environmental factors..."	Omission of formula: This is the most critical deficiency. You define a new metric (S) but do not provide the equation for the "adjustment." How are socio-environmental factors weighted against Net National Income (NNI)? Without an equation, this remains a "black box."
Line 125-127: "The rationalization for this approach... functioning as symbolic capital (K) within the biocultural system."	Conceptual Confusion: You introduce "Symbolic Capital (K)," but in standard economics, K stands for Physical Capital. Furthermore, "symbolic capital" is a sociological term (Bourdieu). Using it in a production function without a mathematical bridge creates a "theory-to-math" gap.
Line 128: "The analysis was conducted using R statistical software."	Incompleteness: Simply naming the software is insufficient. You should provide the version number and a citation for the specific packages used to ensure version control and reproducibility.
Line 130-131: "1. Environment Setup: Integration of readxl... and plm..."	Operational detail: Listing readxl is unnecessary—it is a utility package for data loading. Focus instead on the specific estimators used within plm (e.g., Arellano-Bond, Pooled OLS vs. FE) and how you tested for fixed effects (e.g., Hausman Test).
Line 132-133: "2. Data Merging: The primary socioeconomic and biophysical dataset was merged with the Malmquist Index..."	Endogeneity risk: If the Malmquist Index (an efficiency measure) is merged with its own input variables to predict social well-being, you may have a "circularity" or endogeneity problem. You must explain how you avoided regressing a variable on its own components.
Line 134-135: "3. Model Estimation: We utilized a Fixed-Effects Model to address unobserved heterogeneity..."	Vagueness: You mention "unobserved heterogeneity" but do not mention if you checked for Cross-Sectional Dependence or Autocorrelation, which are standard requirements for long-term panel data (T=30) in *PLOS ONE*.
Line 135-137: "The data was structured as a data_frame, using Country and Year as indices..."	Syntax vs. Methodology: Describing the object as a data_frame is too focused on coding implementation. In a methods section, focus on the Panel Balance (N=6, T=30) and the structure of the error term.
$Yit = \alpha + \beta Xit + \mu i + \varepsilon it$	Variable misalignment: In the abstract, you stated Yit was "Social Well-being." Here, you state Yit represents "Bioeconomic Productivity." This is a significant inconsistency. Is the model measuring what causes productivity, or how productivity causes well-being?

3.3. Analytical Framework: From Traditional TFP to Bioeconomic Productivity (BP)

The bullet point in this section should be numbered 3.3, not 3.2 as currently stated in the manuscript.

3.4. Empirical Estimation: The Malmquist Productivity Index

The bullet point in this section should be numbered 3.4, not 3.3 as currently stated in the manuscript.

Line / Item	Feedback on Shortcomings & Deficiencies
Line 155-157: "The index is decomposed into... EC... and TC... (Eq. 4)."	Standard vs. Novel: This is a standard Malmquist decomposition. The deficiency lies in the claim that TC represents "biocultural innovation." Usually, TC is a residual of the frontier shift. You must explain why, in your model, this shift is *specifically* biocultural rather than general technological progress.

3.5. Econometric Specification and Data Operationalization

- The bullet point in this section should be numbered 3.5, not 3.4 as currently stated in the manuscript.

Line / Item	Feedback on Shortcomings & Deficiencies
Line 168-170: "Following the dimensionality reduction described in the previous section..."	Missing link: You refer to "dimensionality reduction," but sections 3.1–3.3 mentioned normalization and DEA, not dimensionality reduction (like PCA or Factor Analysis). This suggests a missing methodological step or incorrect terminology.
Line 175-177: "Table 2 details the... operationalization of the core variables used to estimate the Social Welfare Function (SWF)."	Conceptual shift: You have suddenly shifted the focus from "Bioeconomic Productivity" to "Social Welfare Function." You must explicitly state if the BP calculated in Eq. 3 is an *input* for the SWF or if they are separate models.
Line 171-173: "Specifically, the transformation of GDP and the Gini coefficient into logarithmic and complementary forms..."	Standardization vs. Innovation: Log-transforming GDP is standard, but you must explain *why* the Gini coefficient is used in its "complementary form" ($1-Gini$). While it represents "Equity," you should cite why this is preferred over the raw index in your specific production function.
Line 173-174: "...and the stability of the long-term equilibrium [40, 41]."	Vagueness: "Stability" in panel data usually refers to the absence of structural breaks or the convergence of the error correction term. You need to specify which econometric test you are using to prove this stability (e.g., CUSUM test).

3.6. Econometric Specification: Fixed Effects (FE) Model

Item	Feedback on Weaknesses & Limitations
Line 179-180: "To validate the transition... adapted Amartya	Theoretical vagueness: "Adapting" a function is not enough for a methods section. You must explicitly state the mathematical

Sen's Social Welfare Function [19, 20]."	form of Sen's function you used (e.g., $W = \mu(1-G)$) before inserting it into a regression.
Line 180-182: "Recognizing the vast disparities... we transitioned from OLS to a Fixed Effects (FE) Panel Data Model [22]."	Methodological gap: You justify FE based on "economic scale," but the standard scientific justification for FE over OLS/Random Effects is the presence of correlation between independent variables and the unobserved error. You must cite a Hausman Test result here.
"This approach significantly improves... (R2) and ensures that the estimated coefficients are not biased..."	Overstatement: FE reduces "omitted variable bias" for static factors, but it does not "ensure" lack of bias. Issues like endogeneity or reverse causality (e.g., does Welfare drive Bioeconomy or vice versa?) remain unaddressed.
Line 182-186: "The use of a log-log specification... stabilized the variance..."	Missing evidence: A log-log specification implies that coefficients are elasticities. You must state if all variables were logged. Also, "stabilizing variance" usually refers to correcting heteroscedasticity; you should confirm if you used "Robust Standard Errors."
Line 194-196: "...leading to an Adjusted R-squared of 0.628, a significant improvement over preliminary OLS models."	Reporting style: In a Methods section, you should describe the *model design*. The actual R^2 results belong in the Results section. Additionally, a higher R^2 does not necessarily mean a "better" model if the underlying assumptions (like stationarity) are violated.

3.7 Model Robustness and Heterogeneity Control

Item	Feedback on Weaknesses & Limitations
Line 198-199: "To ensure the reliability... address the low explanatory power (R2) identified in preliminary tests..."	Weak justification: In econometrics, a low R^2 is not a valid scientific reason to change a model specification. The choice of model should be driven by the underlying data properties (e.g., endogeneity, heteroscedasticity) rather than a desire to increase "explanatory power."
Line 200-202: "First, a log-log specification was adopted for... Biocultural Savings and Real GDP per capita."	Incompleteness: You mention a log-log specification but do not state if all variables were logged. If only two were logged, it is a log-level or log-lin model. Also, you must specify if you checked for unit roots in these logged series (Stationarity).
Line 201-202: "This transformation stabilizes the variance and reduces the impact of extreme values (outliers)..."	Vagueness: While logs can compress outliers, "stabilizing variance" specifically refers to addressing heteroscedasticity. You should explicitly state if you performed a Breusch-Pagan or White test to confirm this improvement.
Line 202-203: "Second, we transitioned from an Ordinary Least	Missing diagnostic: You cannot simply "transition" to FE without providing the result of a Hausman Test. Without

Squares (OLS) estimation to a Fixed Effects (FE) 'Within' Model."	this, the reviewer cannot know if a Random Effects (RE) model would have been more efficient.
Line 204-205: "This approach is crucial for addressing country-level heterogeneity... by controlling for time invariant omitted variables."	Limitation: FE only controls for time-invariant factors. It does not address time-varying omitted variables or simultaneity bias (e.g., does welfare growth drive GDP growth?). This limitation should be acknowledged.
Line 206-209: "These refinements successfully increased the model's explanatory power, yielding an Adjusted R-squared of 0.628..."	Placement error: The specific R^2 value belongs in the Results section. In the Methods, you should describe the *criteria* for model selection, not the final numerical outcome.
Line 207-209: "...indicates that the model now captures more than 60% of the variance... through the proposed bioeconomic pillars [23, 24, 25]."	Conceptual risk: A high R^2 in panel data can sometimes be a sign of spurious regression if the variables are non-stationary. You must prove the variables are cointegrated before celebrating the R^2 value.

Results/ Findings

4.1. Analysis of Bioeconomic Productivity (TFP-AAB)

Line	Feedback on Weaknesses & Limitations
Line 212-213: "The results... reveal distinct trajectories in resource efficiency across the sampled countries."	Vagueness: "Distinct trajectories" is a filler phrase. Specify immediately whether the trend is generally upward (growth) or downward (contraction) for the region.
Line 218-219: "Bolivia and Ecuador exhibit the highest bioeconomic productivity, a growth primarily driven by Efficiency Change (EC)."	Data contradiction: Table 3 shows Bolivia's (tc=1.057) is higher than its pech (1.050). This means growth is driven more by Technological Change, not Efficiency Change.
Line 222-224: "In contrast, Nicaragua (1.008) and Guatemala (1.015) exhibit a profile of active stability..."	Numerical inconsistency: Table 3 lists Nicaragua at 1.221 and Guatemala is not listed with a summary mean in the text. The values in the prose are completely different from the table.
Line 226-231: "Conversely, Mexico and Honduras show average TFP-AAB values below unity (0.972 and 0.985) ..."	Major error: Table 3 shows Mexico at 1.117 and Honduras at 1.010. Both are *above* unity. The text claims they are in "entropic decline" while the data shows productivity growth.
Line 228-231: "According to the data, the rapid increase in CO2 emissions... outweighs the gains in adjusted national income..."	Missing evidence: Table 3 does not show CO2 or water data. You are interpreting results using variables that were not presented in the Results section.

4.2. Heat Map Analysis: The Entropic Flow

Item/ Line	Feedback on Weaknesses & Limitations
Line 257-258: "Figure 2 provides a visual perspective... through the lens of Georgescu-Roegen's Entropy Law [3]."	Conceptual leap: A Malmquist heat map displays productivity change, not thermodynamic entropy. You must clarify the mathematical proxy used to link a TFP > 1 result specifically to "low-entropy" efficiency.
Line 258-260: "The green peaks represent periods where Technological Change (TC) > 1... 'postpone' the entropic limit..."	Theoretical overreach: TC > 1 simply means a frontier shift. Attributing this specifically to "postponing the entropic limit" is a theoretical interpretation that requires proof that these innovations actually reduced energy throughput.
Line 260-263: "Conversely, the red patches reveal declines... likely linked to biophysical pressures or infrastructure deterioration..."	Speculative language: Use of the word "likely" is insufficient for the Results section. You must correlate these "red patches" with specific historical events (e.g., droughts, energy price spikes, or known policy shifts) to validate this claim.
Line 264: "A critical observation is that Efficiency Change (EC) remains near 1.0 across most periods."	Interpretation gap: If EC approx 1, it means countries are staying at the same distance from the frontier (no "catch-up"). You must explain why this implies a lack of "internal optimization" rather than just a stable management baseline.
Line 265-266: "This suggests that... gains are predominantly driven by external technological shifts (TC) rather than an internal optimization of biocultural funds."	Internal contradiction: Your earlier sections argued that AAB (and thus TC) is driven by *ancestral knowledge*. If TC is "external," it contradicts your thesis that indigenous knowledge is a core *internal* driver of the new paradigm.
Line 266-270: "From an entropic standpoint, this highlights a systemic vulnerability... a temporary postponement of the inevitable degradation..."	Deterministic bias: This concludes that the region is failing to build a sustainable model. However, if TC includes "regenerative biocultural innovation," then the degradation is not "inevitable." You must reconcile your entropic pessimism with your "Vivir Bien" optimism.

4.3. Econometric Analysis: From Traditional Welfare to the AAB Paradigm

Consistency: The sample size changes from **174** (Table 4) to **180** (Table 5) without explanation.

Item/ Line	Feedback on weaknesses & limitations
Line 284-285: "To validate the impact... we conducted a two-stage econometric analysis."	Clarity: You must define the "two stages." Usually, this implies 2SLS (Instrumental Variables). If these are just two separate regressions, calling them "stages" is misleading.

Line 285-286: "Initially, we tested the relationship using a proxy for Amartya Sen's Welfare Function (Table 4)."	Operational omission: You never explicitly name the "Proxy" used in Table 4. Is it a composite index? Without a definition, the coefficients are uninterpretable.
Line 292-293: "Similarly, Life Expectancy at Birth is a robust driver of welfare (β = 174.1197, $p < 0.001$)."	Endogeneity risk: If "Life Expectancy" is a predictor of Welfare in Table 4, but becomes the *dependent variable* in Table 5, you have a serious circularity problem that invalidates the two-stage logic.
Line 308-309: "Similarly, the Indigenous Population Index (K) demonstrates a positive impact... (β a = 0.015, $p < 0.1$)."	Weak evidence: A 10% significance level ($p < 0.1$) is generally considered "marginally significant" in *PLOS ONE*. You should avoid describing this as a "demonstrated impact" without acknowledging its fragility.
Line 311-313: "Conversely, the Log Ecological Footprint (CO2) exhibits a substantial negative association... (β = -31.309, $p < 0.01$)."	Magnitude check: A coefficient of -31.3 in a log-log or log-level model is extremely high. This suggests that a 1% increase in footprint reduces life expectancy by 31 years (if Y is years). Check your units and transformations.

Discussion

Item/Line	Feedback on weaknesses & limitations
Line 317-319: "Building upon the preliminary findings... presents a comprehensive analysis using the FE model (Table 5)."	Redundancy: Re-summarizing the transition from OLS to FE for the third time in the manuscript is unnecessary. Move directly to the *implications* of the findings.
Line 320-322: "While the initial proxy model indicated the significance... the FE approach allows us to control country-specific heterogeneity and multicollinearity..."	Technical misconception: Fixed Effects (FE) models control for time-invariant heterogeneity, but they do not inherently control for multicollinearity. Only variable selection or regularization (like Ridge) addresses multicollinearity.
Line 325-326: "The final model reveals that Log Biocultural Savings is a highly significant... predictor (β= 0.685, $p < 0.01$)."	Interpretation error: In a log-log model, the β represents elasticity. You should state that a 1% increase in Biocultural Savings leads to a 0.685% increase in well-being, rather than just calling it a "positive predictor."
Line 326-328: "This finding confirms that the accumulation... translates into improvements... independent of traditional industrial growth."	Overstatement of independence: Since your model still includes GDP as a "strong driver," you cannot claim the effect is "independent" unless you performed a mediation analysis or found GDP to be non-significant.
Line 330-333: "...Log Ecological Footprint, which shows a strong negative pressure... (β= 31.309, $p < 0.01$)."	Sign error: You describe the pressure as "negative" but provide a positive coefficient in the parentheses. This is a confusing typo that contradicts Table 5.

Recommendation

The policy recommendations are high-level/conceptual but lack specific mechanisms for "institutionalizing" non-monetary assets.

Item	Feedback on Weaknesses & Limitations
Line 364-366: "This study demonstrates that the TFP-AAB is a superior metric..."	Overstated claim: "Superiority" is a subjective value judgment. Scientifically, you should state it provides "greater explanatory power" or "reduced omitted variable bias" compared to traditional TFP.
Line 366-368: "By integrating biocultural variables... we have validated that social welfare... does not depend solely on industrial efficiency..."	Generalization: This validation is limited to a sample of 6 countries. You should acknowledge that this finding is specific to the Andean-Amazonian context and may not apply to industrialized bioeconomy.
Line 372-373: "The identification of the 'Biocultural Paradox' constitutes the most significant theoretical contribution..."	Lack of Specificity: You must briefly restate what the paradox is here (e.g., the inverse relationship between short-term growth and long-term fund stability) to make the conclusion self-contained.
Line 374-375: "...confirms it [Biocultural Paradox] is the most stable driver of long-term well-being."	Statistical limitation: "Stable" is a time-series term. Your FE model measures correlation. Without a Cointegration or Unit Root test, you cannot technically claim "long-term stability."
Line 375-379: "Countries like Bolivia and Ecuador... exhibit greater resilience... compared to... Mexico."	Comparative risk: This conclusion is highly sensitive to your small sample size. Ensure the data for Mexico is not being skewed by its significantly larger industrial base, which might require a different normalization method.
Line 381-383: "Through the lens of Georgescu-Roegen's Entropy Law, we have evidenced that 'technological breathers'... are insufficient..."	Conceptual confusion: You are using a thermodynamic law to interpret a productivity index. This "evidence" is metaphorical. Be careful not to claim physical proof where you only have econometric correlation.
Line 383-385: "Persistent entropic pressure... (β= -31.309, $p < 0.01$), acts as a physical brake on longevity, regardless of GDP growth."	Causality vs. Correlation: A negative coefficient does not prove a "physical brake." It proves an association. Furthermore, the magnitude (-31) remains suspiciously high—re-verify if this is an elasticities issue.
Line 389-399: "...regional governments must work to institutionalize biocultural savings by formally recognizing ancestral practices... as core productive assets..."	Implementation gap: There is no mention of how to value these assets. Will you use shadow pricing, contingent valuation, or a non-monetary dashboard? Without a "how," this recommendation is unactionable.

Line 392-395: "This transformation requires a fundamental decoupling of welfare from material throughput..."	Economic reality: "Decoupling" is a highly debated and difficult-to-achieve goal. The recommendation fails to address the potential trade-offs, such as reduced tax revenue or funding for public services that rely on industrial growth.
Line 395-399: "Such a paradigm shift is only sustainable if governments adopt multidimensional metrics..."	Institutional resistance: The conclusion ignores the political and bureaucratic barriers to replacing GDP. Acknowledge that this shift requires significant international cooperation to avoid "capital flight" or credit rating downgrades.
