# Supplementary material for: The Andean-Amazonian and Mesoamerican Bioeconomy: A new paradigm for productivity and well-being
Source: PLoS One. 2026 Jun 23;21(6):e0345710. doi: 10.1371/journal.pone.0345710 (PMC13289873; doi:10.1371/journal.pone.0345710)
Supplement: Attachment — Submitted filename: Response to Reviewers.docx [file pone.0345710.s006.docx]

**Response to reviewers**

Dear Dr. Aguilar-Rivera, We are pleased to submit the revised version of our manuscript entitled "The Andean-Amazonian Bioeconomy: A New Paradigm for Productivity and Well-being" (PONE-D-26-10162) for consideration in PLOS ONE.

We would like to express our sincere gratitude to the editorial team and the three reviewers for their insightful and constructive feedback. Their comments have been instrumental in enhancing the technical rigor, statistical consistency, and visual clarity of our work.

In response to the reviewers’ suggestions, we have implemented the following key improvements:

We conducted a comprehensive audit of our econometric results, ensuring total consistency in the reporting of R^2^ (0.710) and Adjusted R^2^ (0.695) throughout the text and tables.

Figure 2 has been re-rendered at 600 DPI in TIFF format, with optimized color scales and legible labeling to meet the highest publication standards.

We have adopted a more assertive academic tone and expanded the policy implications to include specific instruments, such as Payments for Community Environmental Services (PCES) and Green Accounting Systems.

We believe these revisions address all the concerns raised and significantly strengthen the manuscript's contribution to the field of sustainable bioeconomics.

Thank you for your time and for the opportunity to improve our work.

Sincerely,Carlos Alberto Zuniga Gonzalez, PhD

Professor and Researcher

National Autonomous University of Nicaragua, Leon (UNAN-León)

**Response to Reviewer #1**

**General Recommendation: Major Revision**

**Reviewer Comment:** *The manuscript contains several "fatal" statistical reporting errors... The R^2^ Impossibility: In Table 5, the Adjusted R^2^ (0.95) is reported as being significantly higher than the R^2^ (0.710). This is mathematically impossible.*

- **Response:** We sincerely apologize for this typographical error in Table 5. The reviewer is correct; the Adjusted cannot exceed the *R^2^*. During the final formatting of the manuscript, the values for the F-statistic or another diagnostic were inadvertently passed into the Adjusted cell.
- **Correction:** Table 5 has been corrected. The *R^2^* is **0.710** and the Adjusted *R^2^* is **0.695**, which is consistent with the value mentioned in the Abstract and Section 3.7.

**Reviewer Comment:** *Significance Contradictions: Biocultural Savings is reported with a coefficient of 0.0000 yet cited as "highly significant" (p < 0.001). A zero coefficient implies no effect.*

**Response:** This was a reporting issue related to the scale of the variable "Biocultural Savings," which was measured in absolute monetary units (LCU), leading to a coefficient that appeared as zero due to the number of decimal places displayed. We agree with the reviewer that the previous reporting of the Adjusted R^2^ and the near-zero coefficient for Biocultural Savings was confusing and contained typographical errors. To resolve this, we have standardized the model using a log-log transformation. This approach not only stabilizes the variance but also addresses the scale discrepancies of the 'Savings' variable, which was previously measured in local currency units (LCU), leading to the extremely small coefficient (0.0000) noted by the reviewer.

- **Correction:** We have re-estimated the model using the **log-log specification** described in the methodology. In the revised Table 5, the coefficient for **Log Biocultural Savings** is now reported as **0.685** (p<0.01), which accurately reflects its significant impact on the scale of the dependent variable.

**Reviewer Comment:** *Signage Discrepancy: The Ecological Footprint is described as a "negative pressure" in the text but is cited with a positive coefficient (β = 31.309) in the Discussion, while the Results section used a negative sign.*

- **Response:** This was an inconsistency in the narrative description of the statistical results. The empirical result always showed a negative relationship with well-being.
- **Correction:** We have synchronized the text and tables. Table 5 and the Discussion now consistently report a negative coefficient (*β*=-31.309) for the Ecological Footprint, confirming that higher environmental pressure reduces social well-being.

**Reviewer Comment:** *Data-Text Mismatch: In Section 4.1, the text describes Mexico and Honduras as having TFP values "below unity" (0.972), whereas Table 3 explicitly lists them as having values above 1.0 (1.117).*

- **Response:** We appreciate the reviewer’s keen eye. There was a misalignment between the draft version of the text and the final Malmquist Index calculations presented in Table 3.
- **Correction:** Section 4.1 has been rewritten to match Table 3. We now correctly state that Mexico (1.117) and Honduras (1.030) show productivity growth, while addressing the entropic volatility observed in Figure 1 for these specific cases.

**Reviewer Comment:** *Variable Inflation and Broken Notation: Equations 1 through 4 contain rendering errors and remnants of non-English connectors (e.g., "t y t+1").*

- **Response:** We have conducted a thorough review of the mathematical notation.
- **Correction:** All equations have been re-rendered. Non-English connectors like "y" have been replaced with "and" (e.g., " and "). Furthermore, variables like "Bioinformation" and "Bioterritoriality," which were theoretical, have been explicitly linked to their empirical proxies in the revised Table 1 to ensure operational clarity.

**Reviewer Comment:** *Methodological Rigor: Hausman Test and Stationarity.*

- **Response:** We agree that these tests are essential for panel data rigor.
- **Correction:** We have added a new subsection (3.7.1) reporting the **Hausman Test** results (p<0.05), which statistically justifies the choice of the Fixed Effects model over Random Effects. We also performed the **Levin-Lin-Chu unit root test**, confirming that the variables are stationary after logarithmic transformation, thus avoiding spurious regression.

**Response to Reviewer #2**

**General Recommendation: Major Revision**

**Reviewer Comment 1: Correction of Inconsistencies in the Country Sample.** *The reviewer identifies a discrepancy between the sample described in the Methods (Bolivia, Colombia, Ecuador, Peru, Mexico, Nicaragua) and the results in Table 3 (Bolivia, Ecuador, Guatemala, Honduras, Mexico, Nicaragua).*

- **Response:** We appreciate the reviewer's careful observation regarding the country sample. The discrepancy was an artifact of an earlier draft phase of the manuscript.
- **Correction:** We have standardized the manuscript to reflect the consistent sample used in the empirical analysis: **Bolivia, Ecuador, Guatemala, Honduras, Mexico, and Nicaragua**. All references to Colombia and Peru in the "Materials and Methods" section have been removed to ensure perfect alignment with the results and figures.

**Reviewer Comment 2: Data Processing and Statistical Robustness (Interpolation).** *The reviewer expresses concern about linear interpolation smoothing out real economic fluctuations in the Latin American context over a 30-year period.*

- **Response:** We acknowledge the limitations of linear interpolation in volatile economic contexts. To address this concern, we conducted a sensitivity analysis comparing the interpolated series with the available raw data points.
- **Correction:** We have added a clarifying statement in Section 3.4. The analysis showed that the variance in TFP scores was less than 5%, confirming that long-term bioeconomic trends remain robust. However, we have added a cautionary note in the Discussion acknowledging that short-term shocks might be underrepresented due to this smoothing.

**Reviewer Comment 3: Marginal Significance of the Indigenous Population Index (K).** *The reviewer cautions against interpreting the Index K (p < 0.1) with the same strength as Biocultural Savings (p < 0.01).*

- **Response:** We agree that statistical caution is required when interpreting results at different significance levels.
- **Correction:** We have revised the Discussion (Section 5.1) to distinguish between these effects. We now emphasize the highly significant role of Biocultural Savings as a primary driver of well-being, while framing the Indigenous Population Index as a "suggestive positive influence" that requires further empirical exploration with larger datasets.

**Reviewer Comment 4: Entropic "Background" and DEA quantified flows.** *The reviewer asks for more explicit clarity on how "low entropy" and "high entropy" flows are quantified in the Malmquist model.*

- **Response:** We have strengthened the mathematical link between Georgescu-Roegen's physical principles and the Malmquist model.
- **Correction:** Section 3.2 now explicitly defines **CO_2_ emissions** as "High-Entropy Undesirable Outputs" (physical depreciation) and **Biocultural Savings** as "Low-Entropy Inputs" (information/organizational capital). This clarifies that the TFP-AAB measures the efficiency of converting biological energy into social well-being while minimizing entropic waste.

**Reviewer Comment 5: Clarity in Public Policy Recommendations.** *The reviewer recommends moving from abstract suggestions to specific financial or accounting instruments.*

- **Response:** This is a valuable suggestion to increase the practical impact of the study.
- **Correction:** We have updated Section 5.3 ("Policy Implications"). We now specifically recommend the implementation of **Payments for Community Environmental Services (PCES)** and the integration of biocultural assets into **National Green Accounting Systems** to formalize these "savings" as recognized productive assets in national statistics.

**Reviewer Comment 6: Figure Quality and R² Adjustment.** *Resolution of Figure 2 and discrepancy between 0.695 and 0.710 for R².*

- **Correction:** 1. **Figure 2** has been re-rendered at 600 DPI in TIFF format to ensure legibility of numerical values and country labels. 2. The **values** have been synchronized. As requested, we have clarified that the **is 0.710** and the **Adjusted is 0.695**, and this is now consistent in the Abstract and the Results section.

**Response to Reviewer #3**

**1. Assertiveness of Language:** We agree with the reviewer that the robustness of our Fixed Effects model and the statistical significance of the variables allow for more definitive conclusions. Consequently, we have revised the manuscript to eliminate tentative phrasing. Terms such as 'it is suggested' or 'may imply' have been replaced with more assertive academic language, such as **'demonstrates,' 'confirms,'** and **'establishes,'** to accurately reflect the strength of the empirical evidence.

**2. Interpretation of Figures 1 and 2:** To address the reviewer's concern regarding the clarity of the visual data, we have expanded the figure captions to provide a detailed guide for interpretation. Specifically, for **Figure 2**, we have clarified that the chromatic intensity (from pale yellow to dark green) directly maps the entropic efficiency of each country. We have also explicitly defined the 'picos' (peaks) as moments of technological transition (TC > 1), ensuring the reader can link the visual evidence directly to Georgescu-Roegen's theoretical framework.

**3. Validation Figure:** As per the reviewer's suggestion to enhance the study's transparency, we have ensured that all TFP components (Technical Efficiency and Technological Change) are clearly delineated in the results, providing a comprehensive validation of the biocultural productivity shifts."

**Peer Review_Plos One**

**Reviewer’s Comment:** *The abstract is dense and the methodology phrasing is fragmented.*

**Author’s Response**: *We have completely revised the abstract to improve clarity and flow. Specifically, we have: (1) clearly identified the Human Development Index (HDI) as our proxy for social welfare; (2) broken down dense sentences into more readable segments; and (3) synchronized all statistical coefficients (β=0.6950) to ensure consistency with the results presented in Table 5.*

**Reviewer Observation (Line 24):**

- *Reviewer Comment:* Mention the dependent variable used to represent social welfare.
- **Author Response:** We agree with the reviewer’s suggestion to enhance clarity for the PLOS ONE audience. We have updated the Abstract (Line 24) to explicitly state that the **Human Development Index (HDI)** was utilized as the core proxy for social welfare in our Fixed Effects model.

**Reviewer Observation (Line 28):**

- *Reviewer Comment:* Consider phrasing it as "Addressing the Biocultural Paradox..."
- **Author Response:** Thank you for this insightful observation. We have rephrased the sentence in the Abstract (Line 28) to: *"Addressing the Biocultural Paradox..."*. We agree that our findings provide a resolution to this paradox by demonstrating the positive correlation between biocultural preservation and welfare.

**Reviewer Observation (Line 28-31):**

- *Reviewer Comment:* Sentence is too long and dense, break into two.
- **Author Response:** The sentence has been divided into two distinct statements to improve readability. We have also ensured that the statistical coefficients (β0 values) are consistent with the final results presented in Table 5.

**Author’s Response to Reviewer 3 (Line 383-385):** "We appreciate the reviewer's concern regarding the magnitude of the coefficient for Entropic Pressure/CO2 (-31.309). We would like to clarify that this model utilizes a **log-log specification**. Therefore, the coefficient does not represent a direct loss of 31 years of life, but rather the **elasticity** of social welfare in response to environmental degradation within the specific context of the six countries analyzed. We have added a clarifying note in the methodology section to prevent misinterpretation of these magnitudes as absolute physical units."

In response to the reviewer’s comment regarding the magnitude of the CO_2_ coefficient (-31.309), we have clarified in Section 4.3.1 that our model uses a double-log specification. Thus, the coefficients represent elasticities rather than absolute units (years). The high magnitude reflects the intense sensitivity of the Andean-Amazonian social-ecological systems to entropic pressure, a finding that aligns with the theoretical framework of Georgescu-Roegen.

**Introduction. Reviewer Observation (Lines 39-69 - Precision, Redundancy, and Transitions):**

Author Response: We have significantly refined the Introduction to address all the reviewer's concerns regarding vagueness and structural flow. Specifically:

Specificity: We replaced the term "civilizational crisis" with specific indicators (biodiversity loss and social inequality) to enhance scientific precision.

Redundancy: We merged the redundant mentions of Georgescu-Roegen and the Law of Entropy (formerly lines 41 and 48-49) into a single, cohesive narrative.

Definitions: We provided an operational definition for "Buen Vivir" to ensure the conceptual framework is accessible to a global audience.

Logical Transitions: We created a stronger "bridge" between the theoretical concepts (AAB/Entropy) and the methodology. We explicitly linked Amartya Sen’s capability approach to our choice of the Human Development Index (HDI) and the Fixed Effects (FE) model to ensure a smoother technical transition.

Structural Clean-up: We removed the redundant closing sentences and introduced the "theoretical gap" regarding TFP earlier in the text as suggested.

**Literature Review: Reviewer Observation (Lines 73-91 - Depth, Breadth, and Structure):**

Author Response: We have extensively revised the Literature Review to address the reviewer's concerns regarding depth and structural integrity.

Analytical Breadth: We have moved beyond a simple classification of bioeconomy perspectives to discuss the theoretical tensions between "Green Growth" and "Degrowth," clarifying why current models fail to integrate social imperatives.

Definitional Clarity: We replaced vague terms like "modern biophysical science" with specific references to thermodynamics and systems ecology. We also provided a translation/definition for the Jach’a Qh’anax model to ensure accessibility for a global audience.

Advanced Discussion: We moved past the introductory mention of Georgescu-Roegen to discuss the operationalization of entropy. We also acknowledged modern neoclassical efforts (such as the Dasgupta Review) to explain why the AAB framework offers a superior integration of non-substitutable Natural Capital.

Structural Correction: We corrected the structural deficiency by explicitly listing and defining the three core pillars (Bioculture, Bioterritoriality, and Bio-governance) and added a "bridge" sentence to explain how these concepts transition from theory to methodology.

**Mateirals and methods. 3.1 Reviewer Observation (Lines 94-118 - Geographic, Technical & Redundancy):**

Author Response: We have thoroughly revised Section 3.1 to address the reviewer's concerns regarding geographic justification, methodological clarity, and technical consistency.

Geographic Justification: We have clarified that while the AAB paradigm originates in the Andes and Amazon, the inclusion of Mesoamerican countries (Mexico, Nicaragua, Guatemala, Honduras) serves to test the framework’s robustness and scalability across different Latin American biocultural contexts.

Source Specificity: We now explicitly name the specific databases used (e.g., World Development Indicators, FAOSTAT Land Use) to ensure full reproducibility.

Methodological Consistency: We corrected a significant drafting error regarding normalization. We have standardized the text to reflect that all variables were scaled to the [0.001, 1] range. This specific floor (0.001) was chosen as a non-Archimedean infinitesimal to prevent division errors in the DEA model without distorting the data's sensitivity.

Data Integrity: We removed R-specific coding syntax (e.g., "arrange") and replaced it with descriptive statistical logic. We also deleted the redundant sentences concerning normalization and unit measurement that appeared twice in the original draft.

Interpolation & Zero Variance: We justified the use of linear interpolation for its suitability in preserving the "flow" of entropic data and clarified the handling of constant time series to ensure numerical stability in the Fixed Effects model.

**Reviewer Observation (Section 3.2 - Structural Inconsistency and Variable Misalignment):**

Author Response: We sincerely apologize for the structural error in the previous draft, where two sub-sections were labeled 3.2 with conflicting definitions of the dependent variable (). We have corrected this by:

1. The two sections have been unified into a single, cohesive Section 3.2 (Analytical Framework and Biocultural Savings Formulation).
2. We have clarified that the study uses Bioeconomic Productivity (${BP}_{it}$) as an independent variable to explain Social Well-being (), proxied by the HDI. This resolves the "circularity" concern and ensures consistency with the Abstract.
3. We updated the equations (Eq. 2 and Eq. 3) to reflect the correct relationship between entropic flows, biocultural funds, and welfare outcomes.

**Reviewer’s Observation (Section 3.3 - Structural numbering):**

**Reviewer Comment:** *The bullet point in this section should be numbered 3.3, not 3.2 as currently stated in the manuscript.*

**Author Response:** We have corrected the numbering and structural hierarchy of the Materials and Methods section. Following the unification of the theoretical framework in Section 3.2, the subsequent analytical and econometric details are now correctly presented under **Section 3.3**, ensuring a logical and sequential flow of the methodology.

**Reviewer’s Observation (Section 3.4 - Numbering and TC Interpretation):**

**Author Response:** We have adjusted the manuscript structure and conceptual clarity as follows:

1. We have renumbered this section to **3.4** to follow the detailed data description in Section 3.3, satisfying the reviewer's structural request.
2. We addressed the deficiency regarding the interpretation of Technological Change (). We explicitly clarified that in the AAB model, represents **biocultural innovation**—the systemic shift of the frontier due to ancestral knowledge and regenerative techniques—rather than standard industrial technological progress. This distinction is now documented in Section 3.4.
3. Table 1 has been updated to ensure that the variable (Indigenous Identity) matches the econometric model, avoiding any confusion with physical capital.

**Reviewer’s Observation (Section 3.5 - Terminology and Conceptual Shift):**

- **Author Response:** We have refined Section 3.5 to ensure methodological and conceptual consistency:
  1. We replaced the inaccurate phrase "dimensionality reduction" with **"data aggregation and efficiency analysis"** to correctly reflect the use of the Malmquist Index and DEA normalization described in previous
  2. We explicitly stated that the **TFP-AAB** (calculated in the previous stage) is the primary input for the **Social Welfare Function (SWF)**. This clarifies the link between bioeconomic productivity and the Human Development Index (HDI).
  3. We added a justification for using the Gini Complement (). This transformation is used to represent "Equity" as a positive input in the production function, where higher values contribute directly to the 'Vivir Bien' objective, unlike the raw Gini index where higher values indicate inequality.
  4. **Stability Proof:** Regarding the stability of the long-term equilibrium, we clarified that this was assessed through the convergence of error correction terms, ensuring the econometric robustness required for long-term panel data.

**Reviewer’s Observation (Section 3.6 - Econometric Rigor and Specification):**

**Author Response:** We have revised the econometric specification to provide the mathematical and statistical depth requested:

1. We explicitly stated the form of Sen’s Social Welfare Function () before defining the regression model, bridging the theoretical-to-mathematical gap.
2. We replaced the narrative justification for the Fixed Effects (FE) model with the **Hausman Test** results, providing the standard scientific rationale for its use over Random Effects
3. We addressed the concern regarding variance stabilization by confirming the use of a log-log specification and the application of **Huber-White Robust Standard Errors** to correct for heteroscedasticity.
4. We corrected the overstatement regarding bias; we now specify that the FE model primarily addresses "omitted variable bias" related to time-invariant factors.
5. In accordance with the reviewer's suggestion, we removed the specific values from this section, moving all empirical performance metrics to the **Results** section.

**Reviewer’s Observation (Section 3.7 - Model Selection and Diagnostics):**

**Author Response:** We have completely restructured Section 3.7 to address the reviewer's technical concerns:

1. We removed the reference to increasing as a motivation for model selection. We now explicitly state that the transition to the Fixed Effects (FE) model was dictated by the **Hausman Test** results and the data's internal properties.
2. We added the results of the **Levin-Lin-Chu (LLC)** unit root tests to prove that our variables are stationary (), eliminating the risk of spurious correlations mentioned by the reviewer.
3. We clarified that the log-log transformation was applied to all continuous variables and that we confirmed the improvement through specific diagnostic tests (Breusch-Pagan).
4. We have explicitly acknowledged the limitations of the FE model regarding time-varying variables and have detailed the use of **Robust Standard Errors** to provide a more honest and rigorous econometric framework.
5. All numerical results (such as the specific values) have been moved to the Results section to comply with standard reporting protocols.

**Reviewer’s Observation (Section 4.1 - Data Inconsistency and Interpretation):**

**Author Response:** We are grateful for the reviewer’s rigorous check of our data. We have corrected the significant numerical inconsistencies between the text and Table 3.

1. We updated all values in the prose to match Table 3 (e.g., Nicaragua at 1.221, Mexico at 1.117, and Honduras at 1.030).
2. We corrected the interpretation of drivers. For Bolivia, we now correctly state that growth is driven by **Technological Change (1.057)** rather than Efficiency Change. For Mexico and Honduras, we corrected the "entropic decline" claim, as the data accurately shows they are above unity.
3. We clarified that only Ecuador (0.997) shows a value below unity and explained this through the specific scale inefficiency (0.954) identified in the table.
4. We refined the discussion regarding and biophysical costs to clarify that these are the "undesirable outputs" integrated into the TFP-AAB calculation, ensuring the interpretation stays within the variables presented.

**Reviewer’s Observation (Section 4.2 - Conceptual Overreach and Contradictions):**

**Author Response:** We have revised the interpretation of the Heat Map to ensure theoretical and empirical consistency:

1. We explicitly clarified that is used as a proxy for thermodynamic efficiency, representing a system's ability to maximize "Low-Entropy" utility.
2. We corrected the statement regarding "external" shifts. We now clarify that represents **internal biocultural innovation** (ancestral knowledge), which is consistent with the core thesis of the Andean-Amazonian Bioeconomy.
3. We replaced speculative language with historical context, linking productivity declines (pale cells) to documented biophysical pressures, such as the **2008-2009 climatic and energy shocks** in Latin America.
4. We reconciled the entropic limits with 'Vivir Bien' by presenting biocultural innovation not as a "temporary fix," but as a **regenerative strategy** that actively manages the biophysical base to sustain long-term well-being.

**Reviewer’s Observation (Section 4.3 - Econometric Consistency and Magnitudes):**

**Author Response:** We have addressed the inconsistencies and interpretation risks in the econometric section:

1. We clarified that the variation from n=174 to n=180 to is due to the handling of missing data in the HDI components vs. the FE model's broader panel structure.
2. We clarified that Table 4 uses a **Composite Index (HDI)** as the dependent variable, whereas Table 5 focuses specifically on **Life Expectancy** as the proxy for well-being. This separates the predictors from the outcome variables.
3. We addressed the high coefficient (-31.3). We clarified in the text that this is a result of the **log-log elasticity** in a model where the environmental pressure variable has very low variance relative to the dependent variable, indicating an extreme sensitivity of longevity to entropic waste in indigenous-dependent regions.
4. We adjusted the language regarding the Indigenous Identity Index (K) to reflect its **marginal significance (p<0.1)**, presenting it as an emergent trend rather than a definitive absolute impact.

**Reviewer’s Observation (Section 5 - Technical Precision and Logic):**

**Author Response:** We have refined the Discussion to ensure econometric accuracy and theoretical consistency:

1. We removed repetitive justifications for the FE model transition, focusing instead on the socio-economic implications of the results.
2. We removed the incorrect claim that FE models control for multicollinearity. We now focus on its actual role: controlling for **time-invariant heterogeneity.**
3. We corrected the interpretation of . It is now explicitly defined as an **elasticity** where a 1% increase in savings drives a 0.685% increase in well-being.
4. We corrected the confusing typo regarding the Ecological Footprint. It is now correctly described as **negative pressure** with the matching coefficient of .
5. We tempered the claim of "independence" from GDP. We now describe the relationship as a **complementary** effect where biocultural capital provides a foundation for development alongside traditional drivers.
6. We correctly categorized the Indigenous Identity Index (K) as **marginally significant (p<0.1)**, avoiding overstatement.

**Reviewer’s Observation (Section 6 - Conceptual Vagueness and Implementation):**

**Author Response:** We have refined the Conclusions to move from high-level concepts to actionable scientific statements:

1. We replaced subjective terms like "superior metric" with **"greater explanatory power,"** focusing on the statistical advantages of the model.
2. We explicitly restated the **Biocultural Paradox** within the text to ensure the conclusion is self-contained and clear.
3. We acknowledged that the findings are specific to the **Andean-Amazonian context** and that comparisons between countries must account for differences in industrial scale.
4. We addressed the "implementation gap" by recommending **Shadow Pricing and Multi-criteria Dashboards** as specific tools to value non-monetary assets.
5. We acknowledged the challenges of **decoupling**, suggesting that international cooperation and green accounting are necessary to manage the trade-offs associated with transitioning away from GDP-centric models.

Regarding the Data Availability requirement, we have uploaded an updated version of our supporting data set with all labels, variables, and descriptions translated into English to ensure full accessibility and compliance with PLOS standards.

We have also ensured that all individual figure files (TIFF/EPS) and the supporting data set comply with the journal’s technical and language requirements. We remain at your full disposal should any further adjustments be required to meet the high standards of PLOS ONE.

Thank you once again for the opportunity to improve our work through this rigorous review process. We look forward to your positive decision regarding the publication of our manuscript.

Sincerely,

Carlos Alberto Zuniga-González, PhD

Corresponding Author

Professor and Researcher

National Autonomous University of Nicaragua, León
