# Supplementary material for: The Andean-Amazonian and Mesoamerican Bioeconomy: A new paradigm for productivity and well-being
Source: PLoS One. 2026 Jun 23;21(6):e0345710. doi: 10.1371/journal.pone.0345710 (PMC13289873; doi:10.1371/journal.pone.0345710)
Supplement: Attachment — Submitted filename: Response_to_Reviewers_auresp_2.docx [file pone.0345710.s007.docx]

**Response to reviewers**

May 15, 2026

Dear Dr. Noé Aguilar-Rivera, Academic Editor, PLOS ONE

We would like to express our gratitude to the editorial team and the reviewers for their constructive comments and the opportunity to submit a revised version of our manuscript. We have carefully addressed all the points raised by the reviewers to enhance the clarity, impact, and regional accuracy of our study. Below, we provide a point-by-point response to the reviewers’ comments.

Response to Reviewer #2 (Sergio Gabriel Ceballos Perez)

We sincerely thank Reviewer #2 for his positive evaluation and for identifying himself. His suggestions have been fundamental in strengthening the "heart" of the paper.

1. Interpretation of Elasticity: * Comment: "When using a log-log model, the coefficients represent elasticities... state: 'A 1% increase in biocultural savings translates into a 0.68% increase in well-being (HDI)'."

Response: We agree. We have revised the Results section (Table 5 interpretation) to explicitly state that the coefficient of 0.685 represents elasticity. The text now clearly explains that a 1% increase in biocultural savings translates into a 0.685% improvement in the Human Development Index (HDI).

2. Strengthening the Discussion (The "Biocultural Paradox"): * Comment: "Delving deeper into why it is a paradox... emphasize that the current market penalizes conservation as an 'opportunity cost' while their model demonstrates it is a 'welfare asset'."

Response: We have added a specific paragraph in the Discussion section addressing the "Biocultural Paradox." We now contrast the conventional market view (conservation as an opportunity cost) with our empirical evidence, which frames it as a "welfare asset" essential for long-term productivity.

3. Regional Context and Title: * Comment: "The title mentions 'Andean-Amazonian,' but the sample includes Nicaragua, Guatemala, and Honduras... adjust the title."

Response: We have adjusted the title to "The Andean-Amazonian and Mesoamerican Bioeconomy: A New Paradigm for Productivity and Well-being" to accurately reflect the geographical scope. Furthermore, we have clarified in the methodology that while the study follows the Andean-Amazonian paradigm, the sample includes Bolivia, Ecuador, Guatemala, Honduras, Mexico, and Nicaragua to represent the tropical Latin American belt.

4. Practical Applicability of Theory to Policy: * Comment: "Suggest adding a paragraph on how a real-world decision-maker... could use the Malmquist Index."

Response: We have included a new paragraph in the Discussion section highlighting the utility of the Malmquist Index for policy-makers. We suggest its application for budget reallocation toward biotechnology and ancestral knowledge where the marginal return on well-being is higher.

Response to Reviewer #3

We thank the reviewer for their comments regarding the technical presentation and clarity of the manuscript.

1. Figures/Tables alignment: * Comment: "Ensure all titles in figures/tables align properly, for example efficiency in table 3."

Response: We have reviewed and corrected the formatting and alignment of all tables and figures, specifically focusing on Table 3 as suggested.

2. Glossary/Explanation of technical terms: * Comment: "Include a section where highly technical terms such as 'exogenous' were explained."

Response: We have added a new sub-section titled "Conceptual Definitions" before the Methodology. This section provides clear definitions for key technical terms: Exogenous, Malmquist Index, Production Frontier, and Technological Change.

Final Checks: We have also updated the reference list and ensured that all data availability requirements are met. We believe these revisions significantly improve the manuscript and look forward to your final decision.

Sincerely,

Dr. Carlos Alberto Zúniga-González

Corresponding Author

National Autonomous University of Nicaragua, León.
