# Supplementary material for: The Andean-Amazonian and Mesoamerican Bioeconomy: A new paradigm for productivity and well-being
Source: PLoS One. 2026 Jun 23;21(6):e0345710. doi: 10.1371/journal.pone.0345710 (PMC13289873; doi:10.1371/journal.pone.0345710)
Supplement: Peer review history

# Peer review: Decision Letter 0

**DOI:** 10.1371/journal.pone.0345710.r001

**Authors:** Noé Aguilar-Rivera

15 Apr 2026

PONE-D-26-10162

The Andean-Amazonian Bioeconomy: A New Paradigm for Productivity and Well-being

PLOS One

Dear Dr. Zúniga-González,

Thank you for submitting your manuscript to PLOS ONE. After careful consideration, we feel that it has merit but does not fully meet PLOS ONE’s publication criteria as it currently stands. Therefore, we invite you to submit a revised version of the manuscript that addresses the points raised during the review process.

Please submit your revised manuscript by May 30 2026 11:59PM. If you will need more time than this to complete your revisions, please reply to this message or contact the journal office at plosone@plos.org. When you’re ready to submit your revision, log on to https://www.editorialmanager.com/pone/ and select the 'Submissions Needing Revision' folder to locate your manuscript file.

Please include the following items when submitting your revised manuscript:

- A letter that responds to each point raised by the academic editor and reviewer(s). You should upload this letter as a separate file labeled 'Response to Reviewers'.
- A marked-up copy of your manuscript that highlights changes made to the original version. You should upload this as a separate file labeled 'Revised Manuscript with Track Changes'.
- An unmarked version of your revised paper without tracked changes. You should upload this as a separate file labeled 'Manuscript'.

If you would like to make changes to your financial disclosure, please include your updated statement in your cover letter. Guidelines for resubmitting your figure files are available below the reviewer comments at the end of this letter.

If applicable, we recommend that you deposit your laboratory protocols in protocols.io to enhance the reproducibility of your results. Protocols.io assigns your protocol its own identifier (DOI) so that it can be cited independently in the future. For instructions see: https://journals.plos.org/plosone/s/submission-guidelines#loc-laboratory-protocols. Additionally, PLOS ONE offers an option for publishing peer-reviewed Lab Protocol articles, which describe protocols hosted on protocols.io. Read more information on sharing protocols at https://plos.org/protocols?utm_medium=editorial-email&utm_source=authorletters&utm_campaign=protocols.

As the corresponding author, your ORCID iD is verified in the submission system and will appear in the published article. PLOS supports the use of ORCID, and we encourage all coauthors to register for an ORCID iD and use it as well. Please encourage your coauthors to verify their ORCID iD within the submission system before final acceptance, as unverified ORCID iDs will not appear in the published article. *Only*  the individual author can complete the verification step; PLOS staff *cannot*  verify ORCID iDs on behalf of authors.

We look forward to receiving your revised manuscript.

Kind regards,

Noé Aguilar-Rivera

Academic Editor

PLOS One

Journal Requirements:

When submitting your revision, we need you to address these additional requirements.

1.Please ensure that your manuscript meets PLOS ONE's style requirements, including those for file naming. The PLOS ONE style templates can be found at

https://journals.plos.org/plosone/s/file?id=wjVg/PLOSOne_formatting_sample_main_body.pdf and

https://journals.plos.org/plosone/s/file?id=ba62/PLOSOne_formatting_sample_title_authors_affiliations.pdf

2. Please remove your figures from within your manuscript file, leaving only the individual TIFF/EPS image files, uploaded separately. These will be automatically included in the reviewers’ PDF.

3. Please include captions for your Supporting Information files at the end of your manuscript, and update any in-text citations to match accordingly. Please see our Supporting Information guidelines for more information: http://journals.plos.org/plosone/s/supporting-information.

4. Thank you for providing your underlying data as Supporting Information.

We note that the data set contains text or data that is not in English. Please note that PLOS is an English-language publisher, so we require data sets to be provided in English as well. Please upload an English-language version of your data set.

This will also allow us to determine if your data follows PLOS standards per our Data Availability policy here: https://journals.plos.org/plosone/s/data-availability.

5.If the reviewer comments include a recommendation to cite specific previously published works, please review and evaluate these publications to determine whether they are relevant and should be cited. There is no requirement to cite these works unless the editor has indicated otherwise.

[Note: HTML markup is below. Please do not edit.]

Reviewers' comments:

Reviewer's Responses to Questions

**Comments to the Author**

1. Is the manuscript technically sound, and do the data support the conclusions?

The manuscript must describe a technically sound piece of scientific research with data that supports the conclusions. Experiments must have been conducted rigorously, with appropriate controls, replication, and sample sizes. The conclusions must be drawn appropriately based on the data presented.

Reviewer #1: No

Reviewer #2: Partly

Reviewer #3: Partly

**********

2. Has the statistical analysis been performed appropriately and rigorously?

Reviewer #1: No

Reviewer #2: Yes

Reviewer #3: I Don't Know

**********

3. Have the authors made all data underlying the findings in their manuscript fully available?

The PLOS Data policy requires authors to make all data underlying the findings described in their manuscript fully available without restriction, with rare exception (please refer to the Data Availability Statement in the manuscript PDF file). The data should be provided as part of the manuscript or its supporting information, or deposited to a public repository. For example, in addition to summary statistics, the data points behind means, medians and variance measures should be available. If there are restrictions on publicly sharing data—e.g. participant privacy or use of data from a third party—those must be specified.

Reviewer #1: Yes

Reviewer #2: Yes

Reviewer #3: Yes

**********

4. Is the manuscript presented in an intelligible fashion and written in standard English?

PLOS ONE does not copyedit accepted manuscripts, so the language in submitted articles must be clear, correct, and unambiguous. Any typographical or grammatical errors should be corrected at revision, so please note any specific errors here.

Reviewer #1: No

Reviewer #2: Yes

Reviewer #3: Yes

**********

5. Review Comments to the Author

Please use the space provided to explain your answers to the questions above. You may also include additional comments for the author, including concerns about dual publication, research ethics, or publication ethics. (Please upload your review as an attachment if it exceeds 20,000 characters)

Reviewer #1: Recommendation: Major Revision (Rejection strongly considered)

The manuscript presents an intellectually ambitious and timely framework that attempts to bridge thermodynamic entropy laws with the "Vivir Bien" philosophy through a bioeconomic productivity lens. While the conceptual "Biocultural Paradox" is a compelling contribution to the field, the manuscript currently fails to meet the standards of technical soundness and internal consistency required for publication in PLOS ONE.

The following critical deficiencies must be addressed:

1. Critical Statistical and Mathematical Errors

The manuscript contains several "fatal" statistical reporting errors that undermine the credibility of the empirical analysis:

• The R2 Impossibility: In Table 5, the Adjusted R2 (0.95) is reported as being significantly higher than the R2 (0.710). This is mathematically impossible, as the Adjusted $R^2$ is designed to penalize the addition of variables.

• Significance Contradictions: Biocultural Savings is reported with a coefficient of 0.0000 yet cited as "highly significant" ($p < 0.001$). A zero coefficient implies no effect; if a significant relationship exists, the coefficient must be reported at an appropriate scale or in scientific notation.

• Signage Discrepancy: The Ecological Footprint is described as a "negative pressure" in the text but is cited with a positive coefficient ($\beta = 31.309$) in the Discussion, while the Results section used a negative sign.

2. Internal Inconsistency and Intelligibility

The manuscript suffers from a lack of synchronization between the data tables and the narrative:

• Data-Text Mismatch: In Section 4.1, the text describes Mexico and Honduras as having TFP values "below unity" (0.972), whereas Table 3 explicitly lists them as having values above 1.0 (1.117). This prevents the reader from drawing any valid conclusions.

• Variable Inflation: The methodology introduces several complex variables (e.g., Bioinformation, Bioterritoriality) that are never operationalized in the results, while new variables (e.g., Life Expectancy) appear in the final regressions without prior definition in the theoretical framework.

• Broken Notation: Equations 1 through 4 contain rendering errors and remnants of non-English connectors (e.g., "t y t+1"), making the mathematical logic difficult to parse.

3. Methodological Rigor and Diagnostic Testing

For a 30-year panel analysis, several standard diagnostic tests are missing:

• Hausman Test: The transition to a Fixed Effects (FE) model is mentioned as a "refinement," but the authors provide no statistical justification (Hausman result) to prove FE is superior to Random Effects in this context.

• Stationarity and Spurious Regression: There is no evidence of Unit Root tests (e.g., ADF or Levin-Lin-Chu). Without proving the series are stationary or cointegrated, the high R2 values may simply reflect spurious correlations common in time-series data.

• Endogeneity: The study claims "Biocultural Savings" drives well-being, but fails to address potential reverse causality (i.e., higher well-being leading to better conservation).

Additional Comments

• Ethics: There are no immediate concerns regarding research ethics, provided the authors clarify the source of the "Authors' calculation" for biocultural savings.

• Formatting: The manuscript frequently repeats the justification for the FE model across three different sections. This redundancy should be eliminated to improve conciseness.

• Visual Aids: Table 1 and Table 2 must be reformatted to clearly distinguish between theoretical pillars, empirical proxies, and data sources.

Reviewer #2: 1. Correction of Inconsistencies in the Country Sample

There is a critical discrepancy that must be corrected immediately. While the Materials and Methods section indicates that the sample includes Bolivia, Colombia, Ecuador, Peru, Mexico, and Nicaragua, Table 3 of Results presents data from Bolivia, Ecuador, Guatemala, Honduras, Mexico, and Nicaragua. It is imperative that the authors ensure consistency in the list of countries throughout the text, tables, and figures to avoid compromising the integrity of the findings.

2. Data Processing and Statistical Robustness

Data Interpolation: The study uses linear interpolation and extrapolation to address missing values in a 30-year series (1995–2024). Although this is common practice, over such a long period and in contexts of high economic volatility in Latin America, this could artificially smooth out real fluctuations. A sensitivity analysis is recommended to verify whether the results remain consistent without these interpolations or using more robust imputation methods. Marginal Significance: The impact of the Indigenous Population Index (K) is only significant at the 10% level (p < 0.1). The authors should be more cautious when interpreting this result in the discussion, avoiding presenting this relationship with the same force as biocultural savings, which does show high significance (p < 0.01).

3. Further Exploration of the Entropic "Background" Measurement

The article mentions that the TFP-AAB is defined as a function of entropic efficiency. However, the mathematical transition from Georgescu-Roegen's physical principles to the Data Envelopment Analysis (DEA) model could be more explicit.

Recommendation: Provide greater clarity on how the flow of "low entropy" and "high entropy" (waste) is specifically quantified within the inputs and outputs of the Malmquist model, beyond the general mention of CO2 emissions.

4. Strengthening the Discussion on the "Biocultural Paradox"

The Biocultural Paradox finding is powerful, but it would benefit from a deeper analysis of causality.

Area for improvement: The Fixed Effects (FE) model controls for heterogeneity, but not necessarily for endogeneity (the possibility that greater social well-being, in turn, allows for greater investment in bioculture). The authors could discuss this potential bidirectionality or suggest the use of instrumental variables in future research to confirm the direction of causality.

5. Clarity in Public Policy Recommendations

Although the article recommends "institutionalizing biocultural savings," the suggestions are somewhat abstract. Recommendation: Be more specific about which financial or accounting instruments (e.g., payments for community environmental services, biodiversity bonds, or reforms to the System of National Accounts) would allow these biocultural savings to be formalized as productive assets in government practice.

6. Format and Visualization Aspects

Figure Quality: The Heat Map (Figure 2) has a resolution that makes it difficult to read the specific numerical values by country and year. It is recommended to improve the visual quality of the graphs to facilitate the review of the aforementioned "technological respites." R² Adjustment: The text mentions an adjusted R² of 0.695 in the summary, but then reports 0.710 in the Fixed Effects model results section. This discrepancy should be clarified or corrected.

The manuscript has exceptional conceptual value, but these technical corrections—especially consistency in the country sample and statistical caution—are essential to ensure that its impact on the bioeconomics literature is solid and lasting.

Reviewer #3: This paper presents a large amount of descriptive detail, interesting theories, and statistical values. However the results presented are difficult to interpret: Figure 1 shows plots close to zero with huge peaks at certain years. Figure 2 is very complicated and hard to read. It is not possible to follow the conclusions provided from these two figures. There are too many times in the paper where the words 'is suggested' are used. I recommend that many more figures (with data that is easy to interpret) are required to validate the discussion and conclusions.

**********

6. PLOS authors have the option to publish the peer review history of their article (what does this mean?). If published, this will include your full peer review and any attached files.

If you choose “no”, your identity will remain anonymous but your review may still be made public.

**Do you want your identity to be public for this peer review?**  For information about this choice, including consent withdrawal, please see our Privacy Policy.

Reviewer #1: No

Reviewer #2: **Yes:**  Sergio Gabriel Ceballos-Perez

Reviewer #3: **Yes:**  Floren Plaza

**********

[NOTE: If reviewer comments were submitted as an attachment file, they will be attached to this email and accessible via the submission site. Please log into your account, locate the manuscript record, and check for the action link "View Attachments". If this link does not appear, there are no attachment files.]

To ensure your figures meet our technical requirements, please review our figure guidelines: https://journals.plos.org/plosone/s/figures

You may also use PLOS’s free figure tool, NAAS, to help you prepare publication quality figures: https://journals.plos.org/plosone/s/figures#loc-tools-for-figure-preparation.

NAAS will assess whether your figures meet our technical requirements by comparing each figure against our figure specifications.

Attachment Submitted filename: Peer Review_PLOS One.pdf

# Author comment: Author response to Decision Letter 1

**DOI:** 10.1371/journal.pone.0345710.r002

17 Apr 2026

Dear Dr. Aguilar-Rivera and Reviewers, We have carefully revised our manuscript (PONE-D-26-10162) according to the constructive feedback provided. The main changes are summarized below:

1. Statistical and Technical Rigor:

We corrected the reporting of R2 (0.710) and Adjusted R2 (0.695) in Table 5 to ensure mathematical consistency.

Coefficients were updated to a proper scale (e.g., Biocultural Savings at 0.685) to correctly reflect the elasticity and statistical significance of the variables.

We clarified the transition from OLS (n=174) to a balanced panel for Fixed-Effects (n=180), explicitly citing the linear interpolation used to handle minor missing data.

2. Internal Consistency:

The country sample has been homogenized throughout the entire manuscript (Bolivia, Ecuador, Guatemala, Honduras, Mexico, and Nicaragua).

All signage discrepancies regarding the Ecological Footprint (negative pressure) have been corrected.

3. Theoretical and Policy Framework:

The "Biocultural Paradox" has been restated to be self-contained in the conclusions.

Policy recommendations now include specific instruments such as Shadow Pricing, Multi-criteria Dashboards, and Green Accounting Systems.

4. Editorial Requirements:

Figures have been removed from the main text and provided as high-resolution TIFF files.

The supporting data set has been updated with English-language labels and descriptions.

Captions for Supporting Information have been included at the end of the manuscript.

Please refer to our detailed "Response to Reviewers" file for a point-by-point explanation of all adjustments.

Attachment Submitted filename: Response to Reviewers.docx

# Peer review: Decision Letter 1

**DOI:** 10.1371/journal.pone.0345710.r003

**Authors:** Noé Aguilar-Rivera, Noé Aguilar-Rivera

14 May 2026

PONE-D-26-10162R1

The Andean-Amazonian Bioeconomy: A New Paradigm for Productivity and Well-being

PLOS One

Dear Dr. Zúniga-González,

Thank you for submitting your manuscript to PLOS ONE. After careful consideration, we feel that it has merit but does not fully meet PLOS ONE’s publication criteria as it currently stands. Therefore, we invite you to submit a revised version of the manuscript that addresses the points raised during the review process.

Please submit your revised manuscript by Jun 27 2026 11:59PM. If you will need more time than this to complete your revisions, please reply to this message or contact the journal office at plosone@plos.org. When you're ready to submit your revision, log on to https://www.editorialmanager.com/pone/ and select the 'Submissions Needing Revision' folder to locate your manuscript file.

Please include the following items when submitting your revised manuscript:

- A letter that responds to each point raised by the academic editor and reviewer(s). You should upload this letter as a separate file labeled 'Response to Reviewers'.
- A marked-up copy of your manuscript that highlights changes made to the original version. You should upload this as a separate file labeled 'Revised Manuscript with Track Changes'.
- An unmarked version of your revised paper without tracked changes. You should upload this as a separate file labeled 'Manuscript'.

If you would like to make changes to your financial disclosure, please include your updated statement in your cover letter. Guidelines for resubmitting your figure files are available below the reviewer comments at the end of this letter.

If applicable, we recommend that you deposit your laboratory protocols in protocols.io to enhance the reproducibility of your results. Protocols.io assigns your protocol its own identifier (DOI) so that it can be cited independently in the future. For instructions see: https://journals.plos.org/plosone/s/submission-guidelines#loc-laboratory-protocols. Additionally, PLOS ONE offers an option for publishing peer-reviewed Lab Protocol articles, which describe protocols hosted on protocols.io. Read more information on sharing protocols at https://plos.org/protocols?utm_medium=editorial-email&utm_source=authorletters&utm_campaign=protocols.

As the corresponding author, your ORCID iD is verified in the submission system and will appear in the published article. PLOS supports the use of ORCID, and we encourage all coauthors to register for an ORCID iD and use it as well. Please encourage your coauthors to verify their ORCID iD within the submission system before final acceptance, as unverified ORCID iDs will not appear in the published article. *Only*  the individual author can complete the verification step; PLOS staff *cannot*  verify ORCID iDs on behalf of authors.

We look forward to receiving your revised manuscript.

Kind regards,

Noé Aguilar-Rivera

Academic Editor

PLOS One

Journal Requirements:

If the reviewer comments include a recommendation to cite specific previously published works, please review and evaluate these publications to determine whether they are relevant and should be cited. There is no requirement to cite these works unless the editor has indicated otherwise.

Please review your reference list to ensure that it is complete and correct. If you have cited papers that have been retracted, please include the rationale for doing so in the manuscript text, or remove these references and replace them with relevant current references. Any changes to the reference list should be mentioned in the rebuttal letter that accompanies your revised manuscript. If you need to cite a retracted article, indicate the article’s retracted status in the References list and also include a citation and full reference for the retraction notice.

[Note: HTML markup is below. Please do not edit.]

Reviewers' comments:

Reviewer's Responses to Questions

**Comments to the Author**

1. If the authors have adequately addressed your comments raised in a previous round of review and you feel that this manuscript is now acceptable for publication, you may indicate that here to bypass the “Comments to the Author” section, enter your conflict of interest statement in the “Confidential to Editor” section, and submit your "Accept" recommendation.

Reviewer #2: All comments have been addressed

Reviewer #3: All comments have been addressed

**********

2. Is the manuscript technically sound, and do the data support the conclusions?

The manuscript must describe a technically sound piece of scientific research with data that supports the conclusions. Experiments must have been conducted rigorously, with appropriate controls, replication, and sample sizes. The conclusions must be drawn appropriately based on the data presented.

Reviewer #2: Yes

Reviewer #3: Partly

**********

3. Has the statistical analysis been performed appropriately and rigorously?

Reviewer #2: Yes

Reviewer #3: I Don't Know

**********

4. Have the authors made all data underlying the findings in their manuscript fully available?

The PLOS Data policy requires authors to make all data underlying the findings described in their manuscript fully available without restriction, with rare exception (please refer to the Data Availability Statement in the manuscript PDF file). The data should be provided as part of the manuscript or its supporting information, or deposited to a public repository. For example, in addition to summary statistics, the data points behind means, medians and variance measures should be available. If there are restrictions on publicly sharing data—e.g. participant privacy or use of data from a third party—those must be specified.

Reviewer #2: Yes

Reviewer #3: Yes

**********

5. Is the manuscript presented in an intelligible fashion and written in standard English?

PLOS ONE does not copyedit accepted manuscripts, so the language in submitted articles must be clear, correct, and unambiguous. Any typographical or grammatical errors should be corrected at revision, so please note any specific errors here.

Reviewer #2: Yes

Reviewer #3: Yes

**********

6. Review Comments to the Author

Please use the space provided to explain your answers to the questions above. You may also include additional comments for the author, including concerns about dual publication, research ethics, or publication ethics. (Please upload your review as an attachment if it exceeds 20,000 characters)

Reviewer #2: The work has improved substantially after the first round of revisions: the authors not only corrected obvious mathematical errors (R² and coefficient signs), but also increased methodological rigor by moving from OLS to Fixed Effects and using a log-log transformation. However, solely to improve and ensure the article has the desired impact, I would recommend the following final adjustments:

1. Interpretation of Elasticity: When using a log-log model, the coefficients represent elasticities. The authors should ensure that in the discussion they state: "A 1% increase in biocultural savings translates into a 0.68% increase in well-being (HDI)," rather than referring to absolute units.

2. Strengthening the Discussion (The "heart" of the paper): The "Biocultural Paradox": This is the most powerful finding. I would recommend delving deeper into why it is a paradox. They should be more emphatic that the current market penalizes conservation as an "opportunity cost," while their model demonstrates that it is a "welfare asset."

3. Regional Context: The title mentions "Andean-Amazonian," but the sample includes Nicaragua, Guatemala, and Honduras. I would request a brief explanatory note on why the term is generalized to Central America or, failing that, adjust the title to "Andean-Amazonian and Central American Bioeconomy."

4. Practical Applicability of Theory to Policy: Although they already mentioned "Shadow Prices," I would suggest adding a paragraph on how a real-world decision-maker (e.g., a Minister of Economy in Ecuador or Guatemala) could use the Malmquist Index they calculated to reallocate budgets toward biotechnology or ancestral knowledge.

Thanks a lot for considering the suggestions and working for this paper

Reviewer #3: Ensure all titles in figures/tables align properly, for example efficiency in table 3.

The authors have addressed my recommendations of improved data analysis and presentation of tables and figures.

I do not have the experience in this research area to state that the conclusions are verified by the presented data and its analysis, I need to leave that to the other reviewers.

It would assist the readers if the paper included a section where highly technical terms such as 'exogenous' where explained.

**********

7. PLOS authors have the option to publish the peer review history of their article (what does this mean?). If published, this will include your full peer review and any attached files.

If you choose “no”, your identity will remain anonymous but your review may still be made public.

**Do you want your identity to be public for this peer review?**  For information about this choice, including consent withdrawal, please see our Privacy Policy.

Reviewer #2: **Yes:**  Sergio Gabriel Ceballos Perez

Reviewer #3: No

**********

[NOTE: If reviewer comments were submitted as an attachment file, they will be attached to this email and accessible via the submission site. Please log into your account, locate the manuscript record, and check for the action link "View Attachments". If this link does not appear, there are no attachment files.]

To ensure your figures meet our technical requirements, please review our figure guidelines: https://journals.plos.org/plosone/s/figures

You may also use PLOS’s free figure tool, NAAS, to help you prepare publication quality figures: https://journals.plos.org/plosone/s/figures#loc-tools-for-figure-preparation.

NAAS will assess whether your figures meet our technical requirements by comparing each figure against our figure specifications.

# Author comment: Author response to Decision Letter 2

**DOI:** 10.1371/journal.pone.0345710.r004

19 May 2026

Dear Dr. Noé Aguilar-Rivera, Academic Editor, PLOS ONE

We would like to express our gratitude to the editorial team and the reviewers for their constructive comments and the opportunity to submit a revised version of our manuscript. We have carefully addressed all the points raised by the reviewers to enhance the clarity, impact, and regional accuracy of our study. Below, we provide a point-by-point response to the reviewers’ comments.

Response to Reviewer #2 (Sergio Gabriel Ceballos Perez)

We sincerely thank Reviewer #2 for his positive evaluation and for identifying himself. His suggestions have been fundamental in strengthening the "heart" of the paper.

1. Interpretation of Elasticity: * Comment: "When using a log-log model, the coefficients represent elasticities... state: 'A 1% increase in biocultural savings translates into a 0.68% increase in well-being (HDI)'."

Response: We agree. We have revised the Results section (Table 5 interpretation) to explicitly state that the coefficient of 0.685 represents elasticity. The text now clearly explains that a 1% increase in biocultural savings translates into a 0.685% improvement in the Human Development Index (HDI).

2. Strengthening the Discussion (The "Biocultural Paradox"): * Comment: "Delving deeper into why it is a paradox... emphasize that the current market penalizes conservation as an 'opportunity cost' while their model demonstrates it is a 'welfare asset'."

Response: We have added a specific paragraph in the Discussion section addressing the "Biocultural Paradox." We now contrast the conventional market view (conservation as an opportunity cost) with our empirical evidence, which frames it as a "welfare asset" essential for long-term productivity.

3. Regional Context and Title: * Comment: "The title mentions 'Andean-Amazonian,' but the sample includes Nicaragua, Guatemala, and Honduras... adjust the title."

Response: We have adjusted the title to "The Andean-Amazonian and Mesoamerican Bioeconomy: A New Paradigm for Productivity and Well-being" to accurately reflect the geographical scope. Furthermore, we have clarified in the methodology that while the study follows the Andean-Amazonian paradigm, the sample includes Bolivia, Ecuador, Guatemala, Honduras, Mexico, and Nicaragua to represent the tropical Latin American belt.

4. Practical Applicability of Theory to Policy: * Comment: "Suggest adding a paragraph on how a real-world decision-maker... could use the Malmquist Index."

Response: We have included a new paragraph in the Discussion section highlighting the utility of the Malmquist Index for policy-makers. We suggest its application for budget reallocation toward biotechnology and ancestral knowledge where the marginal return on well-being is higher.

Response to Reviewer #3

We thank the reviewer for their comments regarding the technical presentation and clarity of the manuscript.

1. Figures/Tables alignment: * Comment: "Ensure all titles in figures/tables align properly, for example efficiency in table 3."

Response: We have reviewed and corrected the formatting and alignment of all tables and figures, specifically focusing on Table 3 as suggested.

2. Glossary/Explanation of technical terms: * Comment: "Include a section where highly technical terms such as 'exogenous' were explained."

Response: We have added a new sub-section titled "Conceptual Definitions" before the Methodology. This section provides clear definitions for key technical terms: Exogenous, Malmquist Index, Production Frontier, and Technological Change.

Final Checks: We have also updated the reference list and ensured that all data availability requirements are met. We believe these revisions significantly improve the manuscript and look forward to your final decision.

Sincerely,

Dr. Carlos Alberto Zúniga-González

Corresponding Author

National Autonomous University of Nicaragua, León.

Attachment Submitted filename: Response_to_Reviewers_auresp_2.docx

# Peer review: Decision Letter 2

**DOI:** 10.1371/journal.pone.0345710.r005

**Authors:** Noé Aguilar-Rivera, Noé Aguilar-Rivera, Noé Aguilar-Rivera

9 Jun 2026

The Andean-Amazonian and Mesoamerican Bioeconomy: A New Paradigm for Productivity and Well-being

PONE-D-26-10162R2

Dear Dr.C. A. Zúniga-González

We’re pleased to inform you that your manuscript has been judged scientifically suitable for publication and will be formally accepted for publication once it meets all outstanding technical requirements.

Within one week, you’ll receive an e-mail detailing the required amendments. When these have been addressed, you’ll receive a formal acceptance letter and your manuscript will be scheduled for publication.

An invoice will be generated when your article is formally accepted. Please note, if your institution has a publishing partnership with PLOS and your article meets the relevant criteria, all or part of your publication costs will be covered. Please make sure your user information is up-to-date by logging into Editorial Manager at Editorial Manager® and clicking the ‘Update My Information' link at the top of the page. For questions related to billing, please contact billing support.

If your institution or institutions have a press office, please notify them about your upcoming paper to help maximize its impact. If they’ll be preparing press materials, please inform our press team as soon as possible -- no later than 48 hours after receiving the formal acceptance. Your manuscript will remain under strict press embargo until 2 pm Eastern Time on the date of publication. For more information, please contact onepress@plos.org.

Kind regards,

Noé Aguilar-Rivera

Academic Editor

PLOS One

Additional Editor Comments (optional):

Reviewers' comments:

Reviewer's Responses to Questions

**Comments to the Author**

1. If the authors have adequately addressed your comments raised in a previous round of review and you feel that this manuscript is now acceptable for publication, you may indicate that here to bypass the “Comments to the Author” section, enter your conflict of interest statement in the “Confidential to Editor” section, and submit your "Accept" recommendation.

Reviewer #2: All comments have been addressed

Reviewer #3: All comments have been addressed

**********

2. Is the manuscript technically sound, and do the data support the conclusions?

The manuscript must describe a technically sound piece of scientific research with data that supports the conclusions. Experiments must have been conducted rigorously, with appropriate controls, replication, and sample sizes. The conclusions must be drawn appropriately based on the data presented.

Reviewer #2: Yes

Reviewer #3: Yes

**********

3. Has the statistical analysis been performed appropriately and rigorously?

Reviewer #2: Yes

Reviewer #3: I Don't Know

**********

4. Have the authors made all data underlying the findings in their manuscript fully available?

The PLOS Data policy requires authors to make all data underlying the findings described in their manuscript fully available without restriction, with rare exception (please refer to the Data Availability Statement in the manuscript PDF file). The data should be provided as part of the manuscript or its supporting information, or deposited to a public repository. For example, in addition to summary statistics, the data points behind means, medians and variance measures should be available. If there are restrictions on publicly sharing data—e.g. participant privacy or use of data from a third party—those must be specified.

Reviewer #2: Yes

Reviewer #3: Yes

**********

5. Is the manuscript presented in an intelligible fashion and written in standard English?

PLOS ONE does not copyedit accepted manuscripts, so the language in submitted articles must be clear, correct, and unambiguous. Any typographical or grammatical errors should be corrected at revision, so please note any specific errors here.

Reviewer #2: Yes

Reviewer #3: Yes

**********

6. Review Comments to the Author

Please use the space provided to explain your answers to the questions above. You may also include additional comments for the author, including concerns about dual publication, research ethics, or publication ethics. (Please upload your review as an attachment if it exceeds 20,000 characters)

Reviewer #2: Congratulations to the authors and thank you for your patience in addressing the feedback. The article, from this particular perspective, is ready for publication.

Reviewer #3: (No Response)

**********

7. PLOS authors have the option to publish the peer review history of their article (what does this mean?). If published, this will include your full peer review and any attached files.

If you choose “no”, your identity will remain anonymous but your review may still be made public.

**Do you want your identity to be public for this peer review?**  For information about this choice, including consent withdrawal, please see our Privacy Policy.

Reviewer #2: **Yes:**  Sergio Gabriel Ceballos Perez

Reviewer #3: No

**********

# Editor report: Acceptance letter

**DOI:** 10.1371/journal.pone.0345710.r006

**Authors:** Noé Aguilar-Rivera, Noé Aguilar-Rivera, Noé Aguilar-Rivera

PONE-D-26-10162R2

PLOS One

Dear Dr. Zúniga-González,

I'm pleased to inform you that your manuscript has been deemed suitable for publication in PLOS One. Congratulations! Your manuscript is now being handed over to our production team.

At this stage, our production department will prepare your paper for publication. This includes ensuring the following:

* All references, tables, and figures are properly cited

* All relevant supporting information is included in the manuscript submission,

* There are no issues that prevent the paper from being properly typeset

You will receive further instructions from the production team, including instructions on how to review your proof when it is ready. Please keep in mind that we are working through a large volume of accepted articles, so please give us a few days to review your paper and let you know the next and final steps.

Lastly, if your institution or institutions have a press office, please let them know about your upcoming paper now to help maximize its impact. If they'll be preparing press materials, please inform our press team within the next 48 hours. Your manuscript will remain under strict press embargo until 2 pm Eastern Time on the date of publication. For more information, please contact onepress@plos.org.

You will receive an invoice from PLOS for your publication fee after your manuscript has reached the completed accept phase. If you receive an email requesting payment before acceptance or for any other service, this may be a phishing scheme. Learn how to identify phishing emails and protect your accounts at https://explore.plos.org/phishing.

If we can help with anything else, please email us at customercare@plos.org.

Thank you for submitting your work to PLOS ONE and supporting open access.

Kind regards,

PLOS ONE Editorial Office Staff

on behalf of

Dr. Noé Aguilar-Rivera

Academic Editor

PLOS One
